# Effect of 60 days of head down tilt bed rest on amplitude and phase of rhythms in physiology and sleep in men

**DOI:** 10.1038/s41526-024-00387-3

**Published:** 2024-03-29

**Authors:** María-Ángeles Bonmatí-Carrión, Nayantara Santhi, Giuseppe Atzori, Jeewaka Mendis, Sylwia Kaduk, Derk-Jan Dijk, Simon N. Archer

**Affiliations:** 1 Surrey Sleep Research Centre, Faculty of Health and Medical Sciences, University of Surrey, Guildford, UK; 2 Surrey Clinical Trials Unit, Faculty of Health and Medical Sciences, University of Surrey, Guildford, UK; 3 UK Dementia Research Institute Care Research and Technology Centre, Imperial College London and the University of Surrey, Guildford, UK; 4 Present Address: Chronobiology Laboratory, Department of Physiology, IMIB-Arrixaca, University of Murcia, Murcia, Spain; 5 Present Address: CIBER de Fragilidad y Envejecimiento Saludable, Instituto de Salud Carlos III, Madrid, Spain; 6 Present Address: Department of Psychology, Northumbria University, Newcastle Upon Tyne, UK

**Keywords:** Physiology, Neuroscience

## Abstract

Twenty-four-hour rhythms in physiology and behaviour are shaped by circadian clocks, environmental rhythms, and feedback of behavioural rhythms onto physiology. In space, 24 h signals such as those associated with the light-dark cycle and changes in posture, are weaker, potentially reducing the robustness of rhythms. Head down tilt (HDT) bed rest is commonly used to simulate effects of microgravity but how HDT affects rhythms in physiology has not been extensively investigated. Here we report effects of −6° HDT during a 90-day protocol on 24 h rhythmicity in 20 men. During HDT, amplitude of light, motor activity, and wrist-temperature rhythms were reduced, evening melatonin was elevated, while cortisol was not affected during HDT, but was higher in the morning during recovery when compared to last session of HDT. During recovery from HDT, time in Slow-Wave Sleep increased. EEG activity in alpha and beta frequencies increased during NREM and REM sleep. These results highlight the profound effects of head-down-tilt-bed-rest on 24 h rhythmicity.

## Introduction

Space flight and the associated reduced exposure to gravity are known to cause physiological changes, such as bone and muscle mass loss, cardiovascular system remodelling, and changes in metabolic, immune, mood and cognitive processes. Most of these effects can be mimicked by the head down tilt bed rest (HDBR) protocol (reviewed in ref. 1) which has been widely used by the European Space Agency (ESA) and the National Aeronautics and Space Administration (NASA). Two physiological domains which have not been studied in detail in previous HDBR experiments^2^ nor in space flight studies are the effects on 24-h rhythmicity in physiology and sleep. This may be because either (i) flights have not been long enough, (ii) there were inadequate pre-flight control and post-flight recovery data, or (iii) there have been confounding factors (e.g., different light intensity levels in different parts of the spacecraft)^3^. Nevertheless, there is some evidence that microgravity and space flight have detrimental effects on sleep and 24-h rhythmicity in physiology^3–6^.

Twenty-four-hour rhythmicity is present in physiological variables that relate to several systems and functions such as the cardiovascular system (reviewed in^7^), immune function^8^, metabolism (reviewed in ref. 9) and cognition^10^, which are also affected by microgravity^11–14^. This rhythmicity is generated by the combined effects of rhythms in environmental variables (e.g., temperature and light), rhythms in behavioural variables (e.g., activity, sleep, posture, feeding and fasting), and, importantly, the endogenous circadian timing system.

The endogenous circadian system consists of a hierarchical structure with a main oscillator or pacemaker located in the suprachiasmatic nuclei (SCN) and peripheral oscillators in the rest of the brain and organs and tissues^15^. The phase and the period of the internal circadian timing system is synchronised to rhythms in the environment via entrainment to environmental time signals, or zeitgebers. For humans, the light dark cycle is the main synchronising time signal, although rhythms in physical exercise, social contacts, and meals can also act as zeitgebers. Importantly, in addition to phase, zeitgebers may also impact on the amplitude of rhythmic outputs in physiology, endocrinology and behaviour.

One prominent daily rhythm with profound effects on the autonomous nervous^16,17^, cardiovascular^18–22^ and muscular system^23–27^ is the supine-upright posture cycle, normally associated with the sleep-wake cycle and absent in microgravity even though the sleep-wake cycle persists. These conditions can be modelled by head down tilt bed rest.

Motor activity is also an important circadian output that has been widely studied in humans and allows an indirect evaluation of the sleep-wake cycle^28^. In bed rest conditions, motor activity during the day is lower than in free-living conditions due to the immobilisation and inactivity that accompanies bed rest.

This reduction or absence of rhythmic inputs/outputs feeding back to the circadian timing system is likely to produce a decrease in the amplitude of rhythms in body temperature and hormones^29^ and may also affect the observed timing of these overt rhythms. The latter may be driven by changes in the timing of behaviour or change in endogenous circadian clocks.

Melatonin and cortisol are hormones that follow a circadian pattern and peak at night and in the early morning, respectively. The mechanisms by which these rhythms are driven by the circadian pacemaker are well documented. Based on this mechanistic understanding, the gold standard for assessing the timing of the human circadian pacemaker is the Dim Light Melatonin Onset (DLMO). Other markers may also provide information on the phase of the circadian pacemaker. For example, the timing of the rhythm in wrist skin temperature, which is influenced by both the sleep-wake cycle and the circadian timing system, has been shown to be correlated with DLMO^30^.

Because of the potential negative impact of microgravity or bed rest on circadian rhythms, quantifying these rhythms is of great interest. However, no study has yet measured melatonin or cortisol rhythms in a long-duration bed rest protocol. Furthermore, previous bed rest studies have analysed changes in core body temperature rhythms or in skin vasodilation in response to different stimuli, but no study has performed continuous measurement of peripheral skin temperature. In addition, few studies have quantified the effects of HDBR on sleep structure or the electroencephalogram (EEG) during sleep.

Thus, HDBR, apart from being a good model for the effects of microgravity, is also a protocol relevant to the study of the effects of the removal of the cyclic change in posture on 24-h rhythmicity in the presence of a sleep-wake cycle.

The main objective of this study was to assess the effects of a 60-days HDBR protocol on the phase and amplitude of overt rhythmicity in physiological and behavioural variables. To this end, we assessed melatonin and cortisol rhythmicity in saliva, as well as motor activity, skin temperature, subjective sleepiness and sleep structure and the spectral composition of the EEG during sleep.

## Results

Twenty men successfully completed the protocol, which consisted of three segments: 1) 14 days of baseline data collection (BDC), during which the participants were under a normal posture schedule, 2) 60 days of head down tilt bed rest (HDT) during which the participants were in bed 24 h/day with 6 degrees head down tilt, and 3) 14 days of recovery (R), with identical conditions as BDC. The study was conducted during two campaigns (each enrolling 10 participants), the first occurring January-April 2017 (winter-spring), and the second in September-December 2017 (summer-autumn) (see Methods section for further details).

### Light exposure

**Fig. 1:**
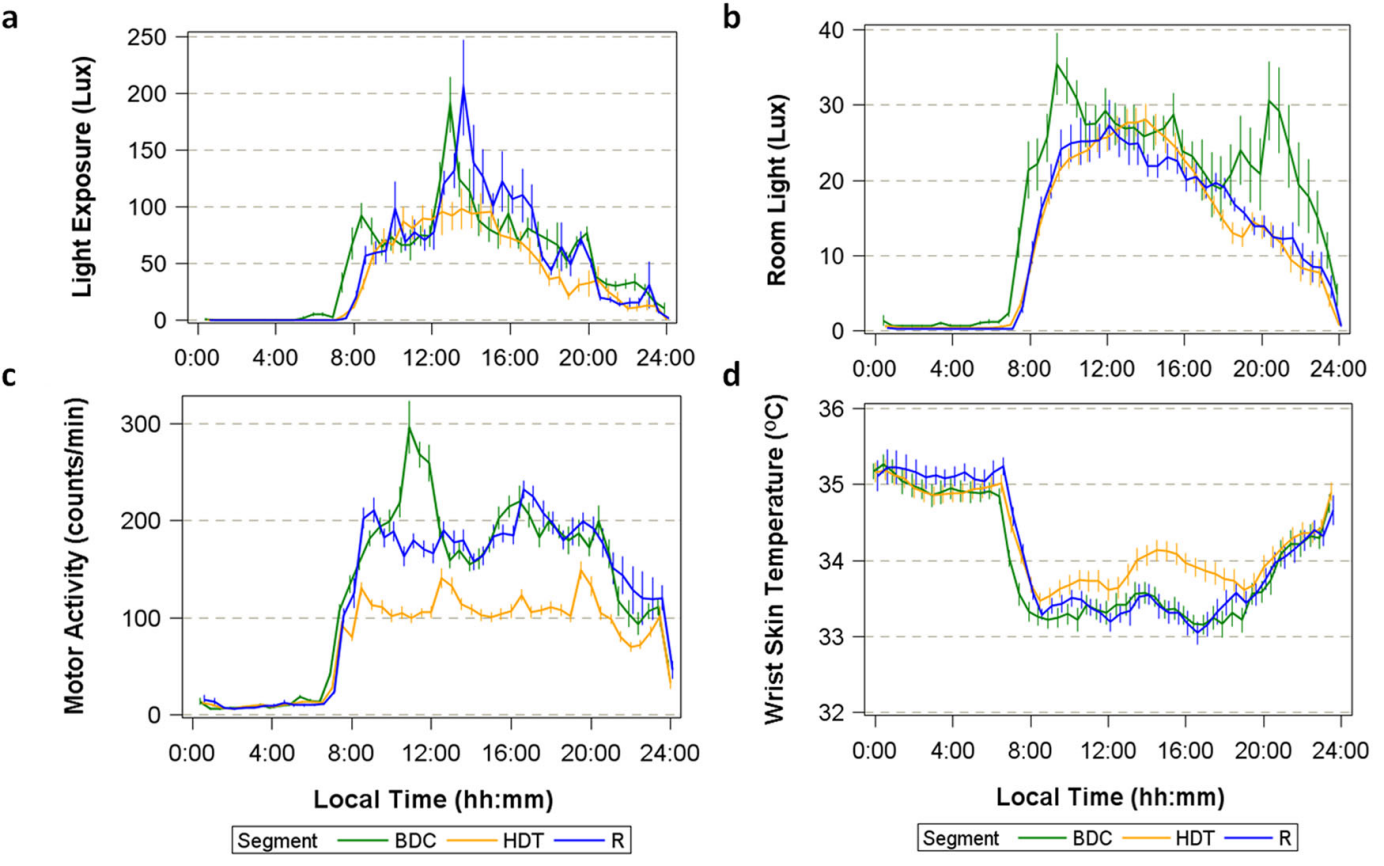
Ambulatory monitoring of light exposure, activity and skin temperature. Average (±SEM) diurnal rhythms of light exposure (personal, **a**; and environmental, **b**), motor activity (**c**) and wrist skin temperature (**d**) assessed through baseline (BDC, green line), head down tilt bed rest (HDT, yellow line) and recovery (R, blue line).

Lights were turned on at ~07:00 and lights-off was scheduled for 23:00 throughout the protocol, although small deviations may have occurred due to clinical needs. Continuous personal light exposure measured at the wrist throughout the entire study demonstrated a reduced and more homogeneous pattern during bed rest (HDT) compared to baseline (BDC) (Fig. 1a). Peaks of high light intensity observed during BDC and Recovery (R) coincided with meals which were served in a dining room during these segments, while the participants remained in the bedroom for meals during HDT. The peaks in light intensity were particularly high during lunch in the BDC and R segments (Fig. 1a). Mixed model analysis revealed a significant effect of ‘segment’ (*p* = 0.0015, see Supplementary Table 1 for statistical details), with the average daytime personal light exposure (08:00–23:00) significantly reduced during HDT (54.87 ± 5.35 SEM lux) compared to BDC (73.18 ± 5.35 lux, p_adj_ = 0.003) and R (71.72 ± 5.22 lux, p_adj_ = 0.0046) (see Supplementary Table 2 for statistical details).

We also recorded environmental room light (Fig. 1b) by means of an Actiwatch device attached to the wall of each bedroom, between the two beds, which yielded, in general, lower light levels than personal light exposure. It should be noted that during BDC and R, participants were allowed to be in different parts of the facilities and not only in the bedroom. Also, during HDT, they were transported to different locations depending on other required tests.

### Motor activity

Motor activity (also continuously measured throughout the study by an actigraph, see Methods) was significantly affected by the protocol (Supplementary Table 1 for statistical details). Although it remained rhythmic, amplitude of activity decreased during HDT (Fig. 1c), due to reduced daytime (08:00–23:00) activity (112.76 ± 5.03 SEM counts per minute) compared to BDC (198.82 ± 5.81 counts per minute, p_adj_ = 0.0003, see Supplementary Table 2 for statistical details) and R (187.97 ± 7.05 counts per minute) (p_adj_ = 0.0003). During BDC, an activity peak was recorded around 10:00 – 12:00, coinciding with scheduled physical exercise (treadmill or bicycle exercises and soft stretching exercises). No physical exercise sessions were scheduled during HDT. Activity peaks that were observed during HDT coincided with mealtimes, since eating in those conditions involved some physical efforts and wrist movements, while during BDC and R, meal time related activity was small compared to activity outside meal times. Night-time activity was not affected by the bed-rest protocol.

### Wrist Skin Temperature

Wrist skin temperature (WST), also recorded throughout the study, showed the expected pattern (Fig. 1d), with higher temperatures during the night and lower values during the day. In general, WST showed a reduction in day-night difference during bed-rest (HDT) (1.17 ± 0.18 °C, SEM) compared to baseline (BDC) (1.63 ± 0.20 °C, p_adj_ = 0.0003, Supplementary Table 2 for statistical details) and recovery (R) (1.78 ± 0.17 °C, p_adj_ = 0.0003). This reduction in the day-night difference was primarily related to a significant increase in averaged daytime WST during bed rest (33.80 ± 0.13 °C in HDT vs. 33.33 ± 0.13 °C in BDC, p_adj_ = 0.0003).

**Fig. 2:**
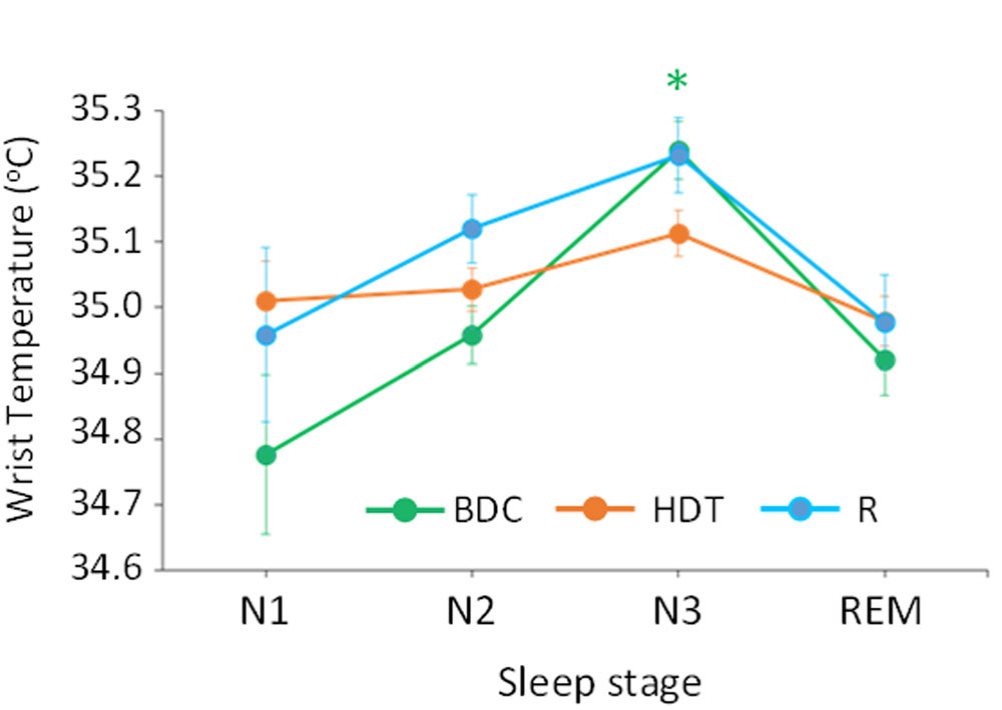
Wrist skin temperature and sleep stages. Mean wrist temperature averaged by sleep stage (N1, N2, N3 and REM) for each protocol segment: baseline (BDC, green line), bed-rest (HDT, orange line) and recovery (R, blue line). Error bars indicate standard error of the mean (SEM). *p_adj_ < 0.0002 for baseline (BDC, green line): N3 vs. N1, N2 and REM; for recovery (R, blue line), p_adj_ = 0.0293, N3 vs. REM. Independent ANOVAs were conducted for each segment, and p-values were adjusted using the Holm-Bonferroni method^78^.

When WST values during sleep were grouped by sleep stage (evaluated through polysomnography during the specific 24 h sampling sessions) (Fig. 2), we found a tendency for WST to increase from non-rapid eye movement (NREM) sleep stages N1 to N3, decreasing again during rapid eye movement (REM) sleep. This tendency was most pronounced during BDC, followed by R, while the difference between sleep stages was clearly reduced during HDT, when none of the sleep stages was significantly different in terms of wrist skin temperature.

### Saliva melatonin rhythms

Saliva for melatonin and cortisol assessment was collected at intervals during specific selected 24-h episodes within the BDC, HDT and R segments. The 24-h sampling episodes occurred twice during BDC (BDC1, days −12/−11; and BDC2, days −4/−3), three times during HDT (HDT1, days 1/2; HDT2, days 26/27; and HDT3, days 53/54), and once during R (days +10/+11) segment. Each sampling episode commenced at 15:00 of the first day (−12, −4, 1, 26, 53 and +10) until 15:00 of the second day (−11, −3, 2, 27, 54 and +11; the sampling sessions of BDC, HDT and R indicates the number of days before the start, or after the start, or after the end of HDT segment, respectively).

**Fig. 3:**
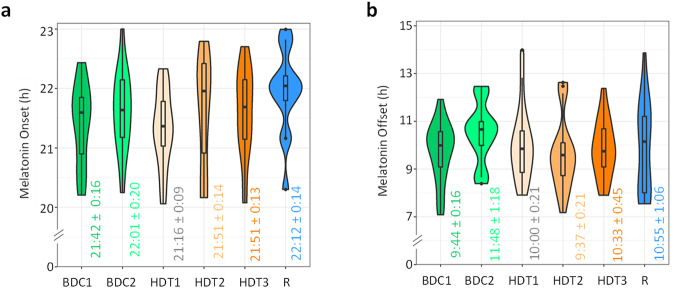
Physiological phase markers. Saliva melatonin onset (**a**) and offset (**b**). Violin plots indicate the data distribution (outline). Horizontal lines indicate median and interquartile range. Mean ± SEM is indicated in text (hh:mm format).

The evening onset rise of melatonin under dim light exposure (dim light melatonin onset; DLMO) is used as a standard marker for endogenous circadian phase. Evening light was not intentionally dimmed to measure DLMO. However, analysis of the evening environmental light levels, as well as recorded personal light participants were exposed to during saliva sampling were low (Table 1). Although we acknowledge the possible influence of this low level of light on the timing of melatonin onset, it is likely not to be relevant in most participants (see Discussion section) (Fig. 3a, Supplementary Table 5).

**Table 1:** Light recorded in bedrooms at wall level, averaged for the 10 rooms (5 each campaign), and personal light exposure at wrist level (*N* = 20 participants) during the evening (18:00 – 23:00) of each sampling session

Sampling session	Evening environmental light during sampling session (lux) (mean ± SEM, *N* = 10)	Evening personal light exposure during sampling session (lux) (mean ± SEM, *N* = 20)
BDC1	36.45 ± 11.31	55.28 ± 12.85
BDC2	19.03 ± 9.53	45.31 ± 7.35
HDT1	16.27 ± 4.15	49.86 ± 18.92
HDT2	14.90 ± 4.63	79.31 ± 31.68
HDT3	12.22 ± 2.44	27.36 ± 10.20
R	10.15 ± 1.29	21.26 ± 3.83

*BDC1* first sampling day during BDC, performed during day -12/-11, *BDC2* second sampling day during BDC, performed during day -4/-3, *HDT1* first sampling day during HDT, performed during day 1/2, *HDT2* second sampling day during HDT, performed during day 26/27, *HDT3* third sampling day during HDT, performed during day 53/54, *R* sampling day during R, performed during day +10/+11. *SEM* standard error of the mean, *N* sampling size.

On average, melatonin onset occurred between 21:15 and 22:15 h and the timing of saliva melatonin rhythms (Supplementary Fig. 1, left panel) was not markedly affected by the protocol. We also analysed melatonin offset (MOff) values (Fig. 3b, Supplementary Table 5). MOff did not show significant differences between the six sampling sessions nor campaigns (p_adj_ > 0.05). However, an interaction between sampling session and campaign was observed for MOn (p = 0.0053, see Supplementary Table 3 for statistical details), indicating an overall tendency to advance in campaign 1 (winter-spring) and a tendency to delay in campaign 2 (summer-autumn) during the HDT segment, potentially due to a possible seasonal effect as a result of campaign separation (see Supplementary Notes, Supplementary Table 5, Supplementary Fig. 2 and Supplementary Discussion).

An effect of the protocol (sampling session) was observed for the evening saliva melatonin levels (averaged over the 18:00–23:00 interval) (Table 2) (*p* = 0.0074, Supplementary Table 3 for statistical details). Evening saliva melatonin levels significantly increased when entering HDT (BDC2 vs HDT1, *p* = 0.0195). In recovery (R), evening saliva melatonin concentrations were significantly lower with respect to the beginning (HDT1) of bed rest (p_adj_ = 0.0324). Morning saliva melatonin concentrations (averaged over the 07:00–12:00 interval), however, did not show a significant effect of sampling session (p_adj_ > 0.05).

**Table 2:** Saliva melatonin concentrations through sampling sessions

Sampling session	Averaged melatonin concentration (pg per mL ± SEM)	Evening melatonin concentration (pg per mL ± SEM)	Morning melatonin concentration (pg per mL ± SEM)
BDC1	4.33 ± 0.44	3.10 ± 0.25	6.23 ± 0.83
BDC2	4.30 ± 0.48	2.51 ± 0.29	6.27 ± 0.87
HDT1	4.57 ± 0.51	3.39 ± 0.38	5.70 ± 0.62
HDT2	5.09 ± 0.66	3.10 ± 0.29	6.01 ± 0.69
HDT3	5.39 ± 0.73	2.94 ± 0.28	6.64 ± 0.91
R	4.46 ± 0.61	2.55 ± 0.25	5.48 ± 0.61

Statistically significant differences in evening melatonin concentration between: BDC2 vs HDT1 (p_adj_ = 0.0195); R vs HDT1 (t = 3.10 df = 77.4, p_adj_ = 0.0324).

Cross-correlation analyses revealed a temporal coincidence (Supplementary Fig. 1, left panel and Supplementary Fig. 2a), between the morning decline (7:00 – 9:00) in wrist skin temperature profile and declining saliva melatonin concentrations, with greater correlation coefficients at the beginning (0.73 ± 0.11, Lag = 0 min) and middle of bed rest (0.70 ± 0.08, Lag = 0 min). The cross-correlation coefficients were lower for the rising part of the curve (Supplementary Fig. 1, left panel and Supplementary Fig. 2b, maximum of 0.57 ± 0.10 for BDC1, Lag = 40 min, WST advanced) compared to the decline in melatonin in the morning, showing a temperature lead in the evening (maximum correlations found at Lags = 40–90 min for all sampling sessions) (positive lags indicate wrist skin temperature profile was advanced with respect to melatonin profile).

### Saliva Cortisol

**Fig. 4:**
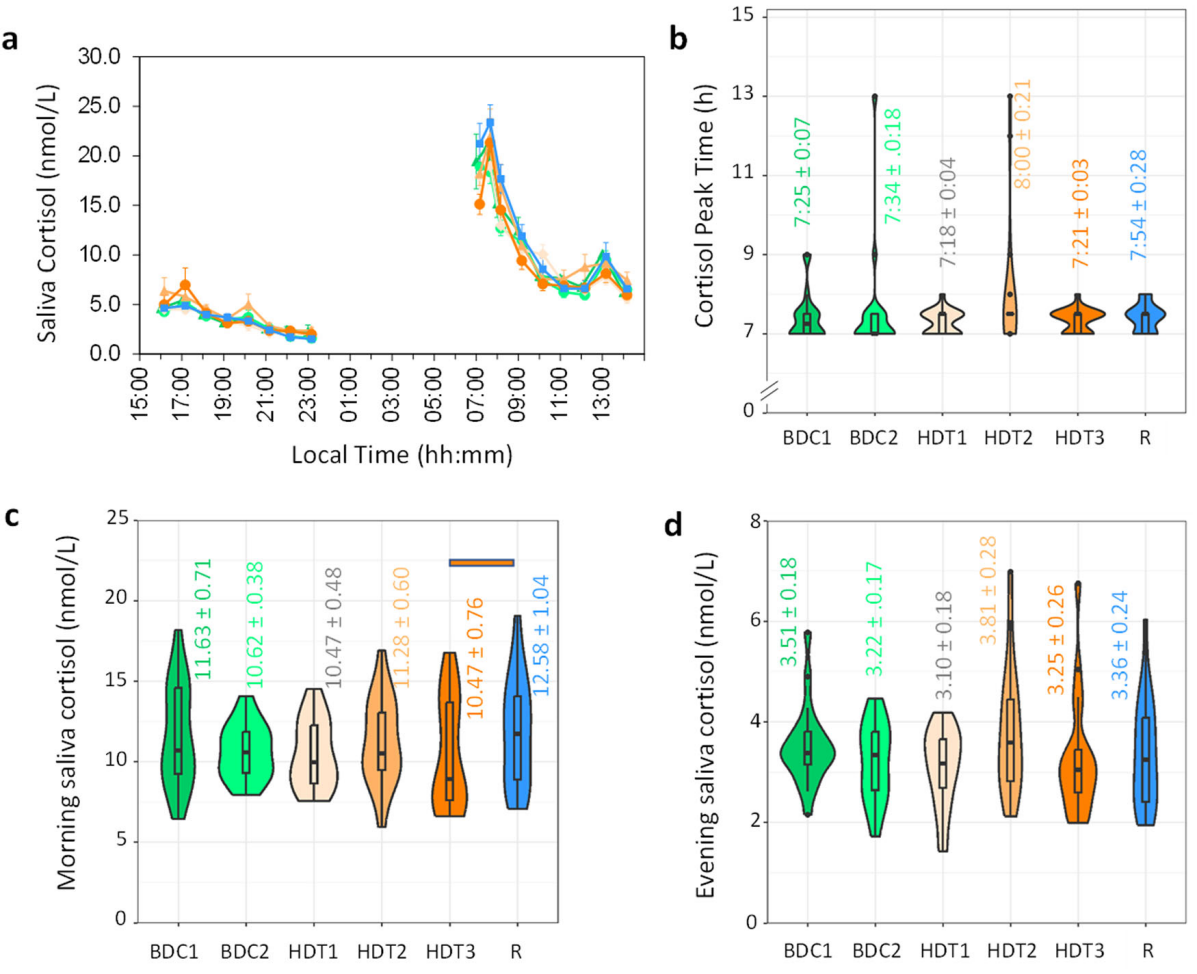
Salivary cortisol. **a** Average (±SEM) salivary cortisol during the sampling sessions (BDC1, dark green; BDC2, light green; HDT1, pink; HDT2, orange; HDT3, red; R, blue); **b** Saliva cortisol peak time; **c** Morning (07:00–14:00) saliva cortisol concentrations and **d** Evening saliva cortisol concentrations (15:00–23:00). Violin plots indicate the data distribution (outline). Horizontal lines indicate median and interquartile range. Mean ± SEM in indicated colour coded text (hh:mm format for peak time). Horizontal colour coded bars indicate statistically significant differences between sampling sessions after Holm-Bonferroni adjustment (p_adj_ < 0.05).

The timing of the cortisol profile in saliva sampled simultaneously with the melatonin samples, did not appear to change across the study (Fig.4a). Average peak cortisol levels occurred between 07:00 and 08:00 during all sampling sessions (Fig. 4b), with no differences between sampling sessions nor campaigns (see Supplementary Table 4 for statistical details). Morning cortisol (07:00–14:00) concentrations increased from HDT (10.74 ± 0.54 nmol per L) to R (12.58 ± 1.04 nmol per L) (p_adj_ = 0.0018), particularly from the last bed rest (HDT3) to recovery (R) sampling session (p_adj_ = 0.031) (Fig. 4c). No differences were found in evening (18:00–23:00) cortisol concentrations (Fig. 4d) (p_adj_ > 0.05).

### Subjective Sleepiness

**Fig. 5:**
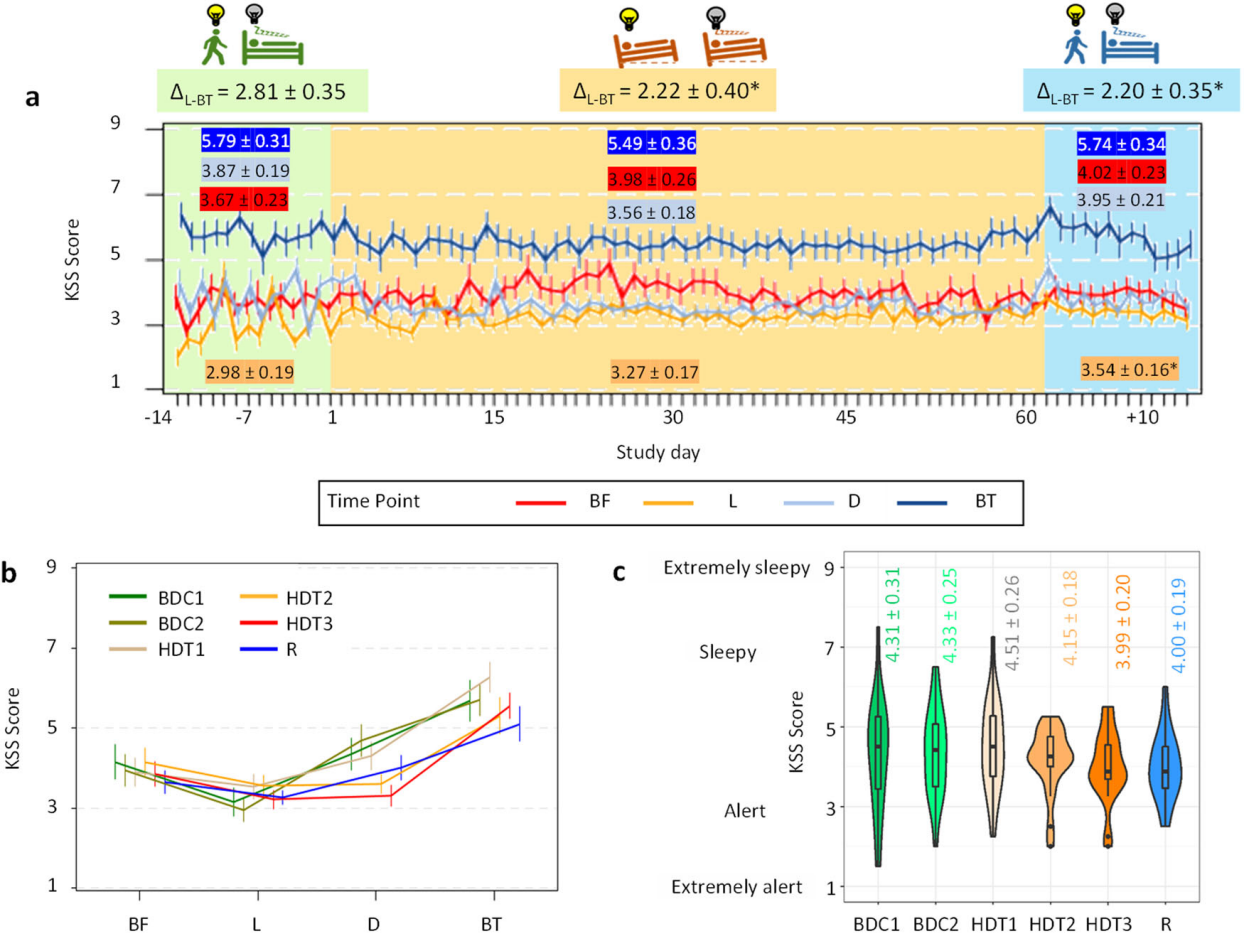
Subjective sleepiness assessed with the Karolinska Sleepiness Scale (KSS). **a** Mean KSS throughout the whole study split by sample time point: before breakfast (BF), lunch (L), dinner (D) and bed time (BT). Error bars indicate standard error of the mean (SEM) and mean ± SEM is colour-coded by time point within each segment. Δ_L-BT_ indicates the difference between sleepiness before lunch and before bed time as a measure of amplitude. * indicates statistically significant differences (p_adj_ < 0.0012) with baseline (BDC); **b** mean waveform by time point (BF, L, D, BT) computed during each sampling session (error bars indicate standard error of the mean (SEM)); **c** KSS score averaged during each sampling session (violin plots indicate the data distribution (outline). Horizontal lines indicate median and interquartile range. Mean ± SEM is indicated in colour coded text format.

Subjective sleepiness was measured every day throughout the study using the 9-point Karolinska Sleepiness Scale (KSS) four times a day: just before breakfast (BF), just before lunch (L), just before dinner (D), and just before bed time (BT) (Fig. 5, Supplementary Table 1 and Supplementary Table 2). Sleepiness scores were higher just before bedtime (BT) throughout the study (Fig. 5a), and during the 24-h previously defined sampling sessions (BDC1, BDC2, HDT1, HDT2, HDT3 and R) (Fig. 5b). Neither mean KSS score (Fig. 5c) nor KSS scores upon awakening, i.e., before breakfast (BF, around 07:00, Fig. 5a) differed across the different segments of the protocol (p_adj_ > 0.05).

The mixed model, which was also applied to these data, yielded the following results (see Supplementary Table 2). Subjective sleepiness just before lunch (around 12:00) was higher in R (p_adj_ = 0.0012) compared to BDC (Fig. 5a). Before dinner (around 19:00), subjective sleepiness tended to decrease during HDT with respect to baseline (p_adj_ = 0.1048), tending to recover during R (p_adj_ = 0.0561). Subjective sleepiness just before bedtime tended to be lower during HDT compared with BDC (p_adj_= 0.1191). In recovery, the values tended to be restored, although the differences with HDT were not statistically significant.

To take a more integrative approach, we evaluated the possible change in the amplitude of subjective sleepiness throughout the study by calculating the absolute difference in KSS scores between lunch and bedtime. This amplitude was significantly greater during BDC than during HDT (p_adj_ = 0.0008) and R (p_adj_ = 0.0006).

### Sleep probability

The probability of being asleep (calculated from dichotomous sleep/wake classification from the EEG) was evaluated during the six sampling sessions (BDC1, days −12/−11; BDC2, days −4/−3; HDT1, days 1/2; HDT2, days 26/27; HDT3, days 53/54; R (days +10/ + 11)) throughout the protocol (Supplementary Fig. 1, right panel). There was a clear rhythmic pattern in sleep probability with maximum values occurring from 23:10 to 07:10. An abrupt decrease in sleep probability was found at 03:00 due to the blood sampling (performed by clinical staff using a red headlight), scheduled as part of the protocol. Most participants went back to sleep within a short period of a few minutes. A second peak of sleep probability was observed from 13:10 to 14:00, when participants were required to remain quiet (seated during baseline and recovery, or just quiet and as immobile as possible during bed-rest) in order to acquire a movement-artefact free daytime EEG signal. Some of the participants fell asleep during that period (which they were allowed to), which is reflected as a diurnal peak of sleep probability.

We also performed cross-correlations analyses between wrist skin temperature and sleep probability (Supplementary Fig. 1, right panel and Supplementary Fig. 2c), showing a strong temporal coincidence (Lag = 0 min and correlations coefficients of 0.61 ± 0.06 maximum for BDC1; 0.54 ± 0.07 maximum for HDT at HDT3; and 0.50 ± 0.06 for R sampling session) (positive lags indicate wrist skin temperature profile was advanced with respect to sleep probability profile).

### Nocturnal sleep parameters

**Fig. 6:**
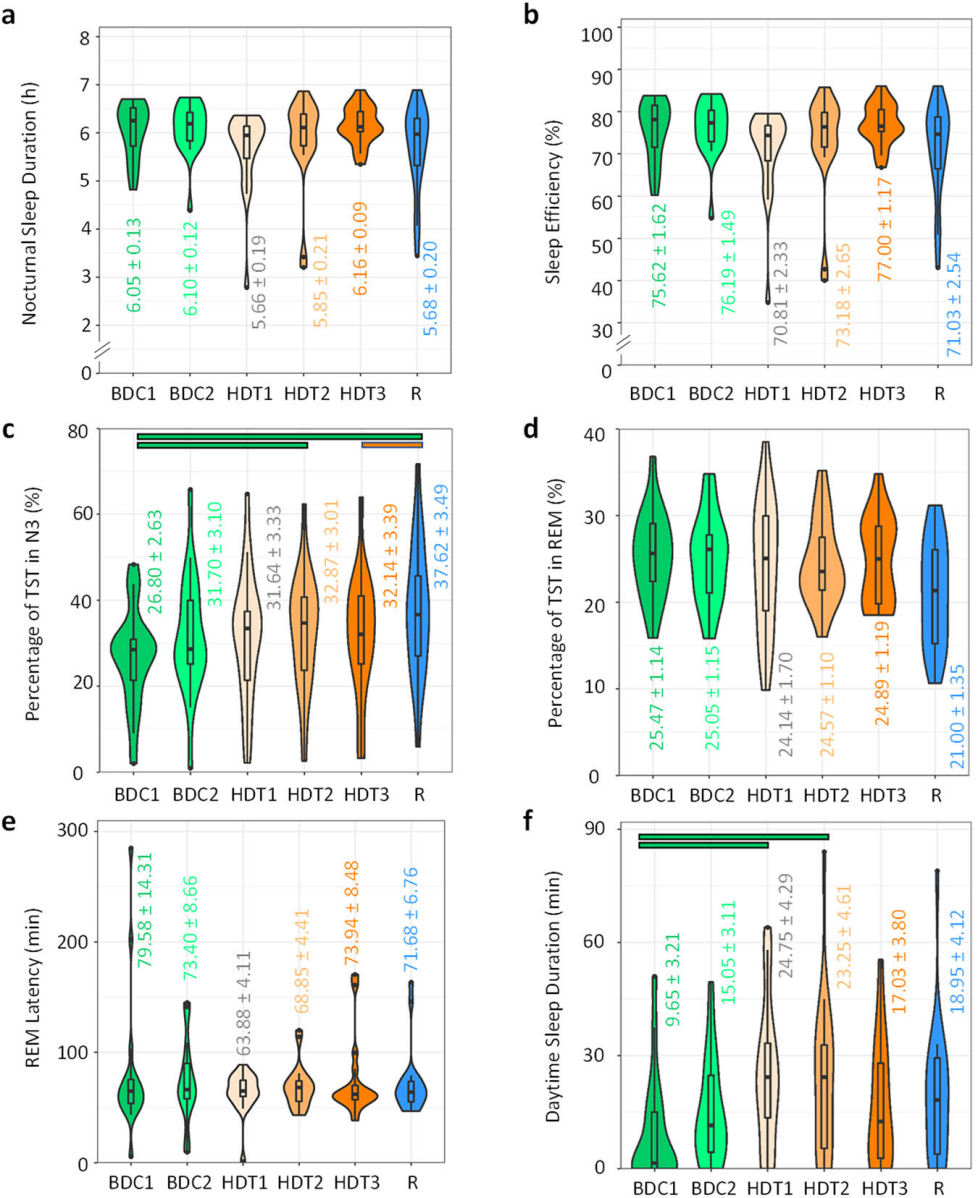
Sleep parameters during the protocol segments. Nocturnal sleep duration (**a**), sleep efficiency (**b**), percentage of total sleep in N3 (**c**) and REM (**d**), REM latency (**e**) and diurnal sleep duration (**f**). Violin plots indicate the data distribution (outline). Horizontal lines indicate median and interquartile range and the mean ± SEM is colour-coded for each segment and variable. Horizontal colour coded bars indicate statistically significant differences between sampling sessions after Holm-Bonferroni adjustment (p_adj_ < 0.05).

The maximum overall average PSG assessed night-time sleep duration was 369.58 ± 5.63 min (6.16 ± 0.09 h) (Fig. 6a) with the maximum duration occurring in the last sampling night of bed rest (HDT3). Although no significant effect of the protocol was found, the overall tendency was for average night-time sleep duration to decrease from baseline to the very first day of bed rest, then progressively increase through bed rest, and decreasing again during recovery. As expected, the same tendency was observed for sleep efficiency, i.e., total sleep time as expressed as a percentage of the lights off period, with lowest values of 70.8% during HDT1 and 77% during HDT3 (Fig. 6b).

While the percentage of time spent (i.e., sleep stage as a percentage of total sleep time) in N1 and N2 did not significantly change throughout the protocol, participants spent a higher proportion of time in N3, with an average of 32.87% during HDT2 and of 37.6% during the recovery segment (R) compared to 26.8% in BDC1 (p_adj_ < 0.0242, see Supplementary Table 4 for statistical details) and approximately 32% during HDT sampling sessions (HDT3 vs. R, p_adj_ = 0.0048) (Fig. 6c). By contrast, the percentage of time spent in REM (Fig. 6d) did not significantly change across sampling sessions, although when computing over the entire segment, during R, participants seemed to spend a lower percentage of time in REM than in BDC (p_adj_ = 0.0462). REM latency values were within a normal range (Fig. 6e) and tended to decrease at the beginning of bed rest in comparison with baseline, to progressively recover throughout the bed rest segment, to decrease again in recovery (R) (differences were not statistically significant).

### Daytime sleep duration

Sleep during the day (naps), which were allowed and assessed through PSG (Fig. 6f), showed an inverse pattern compared to night time sleep and the effect of the HDT was significant compared to BDC (p_adj_ = 0.0069), with an abrupt increase at the beginning of bed rest (HDT1) compared with BDC1 (p_adj_ =0.0078), which lasted until the middle of the bed rest segment (HDT2 vs. BDC1, p_adj_ = 0.024), to then progressively decrease reaching similar levels to baseline at HDT3 (p > 0.05). Daytime sleep duration during recovery was similar to the end of bed rest.

### Quantitative analysis of the EEG

**Fig. 7:**
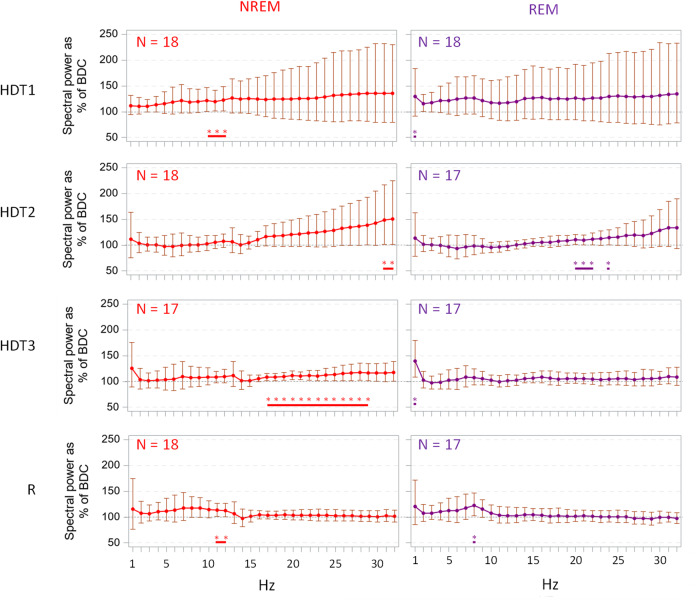
EEG power density. EEG power density during bed rest (HDT1, HDT2, HDT3) and recovery (R1) during NREM (left) and REM (right) sleep (unlogged geometric mean and 95% confidence intervals) averaged across entire night and expressed relative to baseline (BDC) (*p* < 0.05).

Quantitative analysis of the EEG signal averaged over the whole night (Fig. 7) revealed higher spectral power in higher frequencies such as alpha and beta in NREM during bed rest (HDT1 10–12 Hz; HDT2 31–32 Hz; HDT3 17–29) and recovery (R1, 11–12 Hz) compared to baseline. During REM sleep, there were significant increases during bed rest in a few rather narrow frequency ranges (HDT1 1 Hz; HDT2 20–22, 24 Hz; HDT3 1 Hz), as well as in recovery (8 Hz).

## Discussion

We present a comprehensive analysis of biological rhythmicity and sleep physiology performed during all segments of a head-down tilt bed rest (HDBR) protocol. This included the study of inputs to the circadian system (e.g., light exposure), as well as outputs with smaller (e.g., motor activity) and more prominent endogenous circadian components, such as melatonin and cortisol secretion and wrist skin temperature rhythms. In general, timing of rhythms did not change throughout the study, but most of them showed a reduced amplitude during HDBR compared to the baseline and recovery.

In microgravity and during head down tilt bed rest, the strength of the rhythmic environmental, behavioural and physiological feedback to endogenous rhythmicity is reduced. This may lead to reduced strength of overt rhythmicity. In our study, a reduction of amplitude during HDBR was observed in wrist activity due to the confinement and movement restrictions, similar to Liang et al. ^31^ We also found a decrease in the amplitude of wrist skin temperature. In line with our results, other authors have found a reduction in the amplitude of body temperature rhythms under HDBR conditions^32^ and space flight^3,33^. Similar to our wrist skin temperature data in HDBR, Gundel et al.^4^, reported that body temperature during the night was higher in space^4^, which, according to the authors, might be an indication of reduced circadian amplitude. Other studies, however, failed to detect any effect of HDBR on the amplitude of core body temperature^34,35^.

The reduced amplitude in wrist skin temperature could be due to a shift in the parasympathetic/sympathetic balance associated with bed rest that results in peripheral vasodilatation (reviewed in^36^). Also, the post-prandial increase of peripheral temperature was more pronounced during bed rest, which could have also induced enhanced sleepiness during that period^37^. Subjective sleepiness in our study was higher just before lunch during bed rest and remained significantly higher during recovery compared to baseline. Also the amplitude in sleepiness scores, which is closely related to body temperature^32,38^, was reduced during bed rest. Previous results from head-down and horizontal bed rest studies showed an absence of diurnal variation in sleepiness^3^, which is in contrast to the diurnal variation in sleepiness during normal conditions, where an increase in sleepiness in the evening is observed^39^.

In our study, evening melatonin concentrations increased during bed rest, contrary to the effects of acute posture change from upright to supine position previously found^40,41^. We cannot discard the possibility that the lower levels of light exposure during that segment produced that increase. Morning cortisol concentrations increased during recovery after bed rest, whereas Dijk et al.^3^ found a trend for cortisol to increase during space flight, and decrease after flight^3^. Other authors reported a reduction in cortisol during bed rest and no changes in melatonin profile^42,43^. Previous studies also found a reduction in the amplitude of cardiovascular rhythmic variables such as heart rate^31^ or systolic blood pressure^44^ under bed rest conditions. However, although changes in the amplitude of diurnal rhythms are evident in different body functions during bed rest, it should be considered that this reduction can be attributed to inactivity and confinement and not necessarily to the effects of microgravity simulation^45^.

In our study, the timing of saliva melatonin and cortisol secretion and other rhythmic variables did not change throughout the experiment, which is not surprising considering the stable light-dark cycle, albeit with reduced strength. Although other authors have found a significant phase delay in core body temperature during bed rest conditions^34,35^, others found only minor^46^ or non-significant changes in circadian phase in this and other rhythmic variables^42,43,47–49^. Other variables, such as heart rate, have been observed to exhibit delays in bed rest conditions^31^. It should be noted that previous experiments showing significant phase shifts under HDBR conditions also involved the absence of daylight exposure^32^, which could explain this effect. In our study, the strength of the light-dark cycle tended to be reduced (<50 lux environmental light measured in bedrooms) during bed rest, although in this case we did not find any evidence of phase shifts. Regarding the possible limitations of our melatonin onset parameter, although the light was not intentionally dimmed for saliva melatonin sampling, the environmental levels recorded during those sampling evenings ranged from 10 to 36 lux. Although we cannot discard the presence of participants highly sensitive to light, according to published data, these light intensities are unlikely to have produced relevant melatonin suppressions^50,51^. Furthermore, the average timings of melatonin onset (21:16 to 22:12) are within the normal range of previously published DLMO timings^52–54^.

Studies conducted in space^4,33^ found body temperature to be delayed by about 2 h during spaceflight. Dijk et al. ^3^ also reported a potential delay in cortisol rhythms with respect to sleep timing during late flight but is important to note that in this study the period of the imposed sleep-wake cycle was 20–35 min shorter than 24 h^3^.

This is the first time that systematic sleep monitoring has been carried out in a prolonged bed rest study^1^. Sleep duration was short (<6.5 h) throughout the entire study, similar to durations previously recorded in other bed rest^55^ and space studies^3,6,56,57^, and also recorded in the elderly^29^ (and reviewed in^58^). Partial sleep deprivation of a similar extent has been found to be relevant for changes in immune system function and inflammatory status during both bed rest and spaceflight^59–61^, and for immune suppression observed in ageing (reviewed in^62^). Indeed, just one week of restricting the sleep opportunity to 6 h has been shown to disrupt the expression of immune function related transcripts in human blood cells^63^.

A −12^o^ HDBR study found no reduction in estimated sleep duration but did record a decrease in subjective sleep quality^64^. This may have been explained by an associated reduction in slow wave sleep^65^, which has also been reported in a study by Nday et al. ^64^, but contrary to results that showed an increase in deep sleep during a horizontal bed rest protocol^66^. In our protocol, we found an increase of N3 during recovery, while REM only tended to be reduced during that segment. In line with these results, Dijk et al. observed an increase in REM sleep after return to earth^3^. This change may reflect an increased sleep homeostatic pressure resulting from the period spent in HDBR.

The EEG results showed a significant increase in spectral power in the alpha band when entering HDBR and also when regaining the normal posture cycle during recovery, while spectral power of beta frequencies increased as HDBR progressed. As far as we know, this is the first time that spectral analysis of the sleep electroencephalogram has been performed in these prolonged HDBR conditions. Other studies found similar results in wake EEG, with alpha and beta bands increased under HDBR^67^, while other authors found a decrease in mean EEG spectral power (also in wakefulness) after the onset of HDBR, remaining decreased during HDBR, and returning to baseline levels in recovery^68^. In our study we did not observe changes in the hallmarks of NREM sleep, i.e., slow waves and sleep spindle activity and this is in contrast to the results of quantitative analysis of the sleep EEG studied during space shuttle missions which revealed changes in sleep spindle activity and aspects of EEG slow waves^69^.

As expected, wrist skin temperature was higher during the night and lower during the daytime, due to the alternating balance between parasympathetic (vasodilation) and sympathetic (vasoconstriction) actions on peripheral skin vessels, driven in part by the central SCN clock^70–72^ and also potentiated by the sleep-wake cycle^73^. In our study, this rhythm was found to be coincident and correlated with sleep probability and saliva melatonin concentrations^30^, even in bed rest conditions, reflecting its predictive capacity for other rhythms. Also, it should be noted that temporal coincidence was greater when considering the morning melatonin decrease rather than the evening melatonin increase, when a lead of wrist skin temperature was detected, in line with previous results^30^. Although the light levels recorded during saliva sampling should not be expected to produce strong melatonin suppression^50,51^, we cannot exclude that the evening increase of melatonin concentrations was in part suppressed by light. Also, since our saliva sampling protocol did not permit us to capture the peak melatonin concentration, the decreasing phase of the melatonin profile (which occurs in the morning) may exhibit a stronger correlation with this parameter than the onset phase.

Regarding the relationship between thermoregulation and sleep in this protocol, during baseline, wrist skin temperature increased from NREM1 to NREM3 and decreased again in REM, consistent with published data^74–76^, demonstrating the tendency for peripheral temperature to increase as sleep gets deeper and the masking effect of sleep on this rhythm. This relationship, however, was partially abolished during bed rest, when the wrist temperature differences between sleep stages almost completely disappeared. Despite the masking effects of activity, etc., the correlations between wrist skin temperature and sleep probability and saliva melatonin confirm its usefulness as a marker of rhythmicity that can be used in conditions when other measurements are not easy to perform, such as in spaceflight or the elderly.

In addition, some seasonal effects (resulting from the separation of the study into two campaigns and detailed in SI) were detected in wrist skin temperature, motor activity, light exposure (Supplementary Fig. 3, Supplementary Fig. 4), subjective sleepiness, melatonin (Supplementary Figs. 5, 6; Supplementary Table 5; Supplementary Table 6) and sleep duration (Supplementary Notes, Supplementary Discussion).

This protocol provided some interesting data on 24-h rhythmicity in a number of physiological variables. However, and probably because HDBR have been traditionally designed for non-circadian studies, we must acknowledge some limitations. An important potential confounding factor to consider when studying biological rhythms and sleep is light exposure and this was not the same between campaigns, nor across the different sampling segments of the protocol. This may have contributed to some of the observed differences in dependent variables between segments, although not within them. Participants were also allowed to use screen devices (e.g., laptops, tablets, smartphones, etc.), which may have also affected some variables as well as sleep parameters. Thus, the standardisation of light exposure is an outstanding issue in bed-rest studies, as well as in spaceflight^3^. Another limitation was the absence of intentional dimming light exposure in the evening in order to get a proper melatonin phase marker (i.e., dim light melatonin onset, DLMO). However, although the information provided cannot be considered a DLMO, we believe our melatonin onset can be informative about the participants’ circadian status. According to published data, the recorded room light (10 – 36 lux) during the sampling evenings are unlikely to have produced relevant melatonin suppressions^50,51^, although we cannot discard the possibility of some participants being especially sensitive to light^50,51^. Besides, the average timing of our MOn (21:16 to 22:12) is within the normal range of previously obtained DLMO timings^52–54^. Also, the history of sleep patterns was not taken into account during recruitment (see Supplementary Table 7). It should be noted, however, that the previous sleep schedules closely resembled the clinic schedule, and there were only minor variations in participants’ sleep schedules before entering the study (average bed time, 23:08 ± 00:44, mean ± SD; average wake up time 07:10 ± 01:02, Supplementary Table 7), so any potential effects are expected to be modest. In addition, participants’ sleep was disturbed for the one night time blood sample, which may have affected the validity of the sleep duration measures, although the sleep measures expressed as percentage of total sleep time should be considered valid. Another limitation of this study is the exclusion of female participants and the limited age range of the participants, so it may be premature to extrapolate our conclusions to a more diverse population.

Regardless of these limitations, this protocol provided an opportunity to perform a comprehensive study on biological rhythms and sleep in a long-duration head down tilt bed rest protocol. The data show reductions in the amplitude of most of the rhythms studied, as well as an impairment of sleep quality. Our results highlight the importance of monitoring sleep and biological rhythms during simulation studies and also spaceflight, and emphasise the need for interventions to counteract the negative impact of sleep and circadian disruption.

## Methods

### Participants

Twenty healthy male volunteers (age 20–45, 34.15 ± 7.63, mean ± SD) participated in this study, which was approved by the Comité de Protection des Personnes SUD-OUEST ET OUTRE-MER I, and by the Agence nationale de sécurité du medicament et des produits de santé. Written informed consent was obtained from each participant prior to the study after the participants had been fully informed of the study details. Approval was obtained for the transfer and storage of samples at the University of Surrey. Recruited participants did not suffer from a sleep disorder (based on medical history) and did not perform shift work and had not travelled across more than 1 time zone in the 2 months prior to starting the study.

### Protocol

**Fig. 8:**
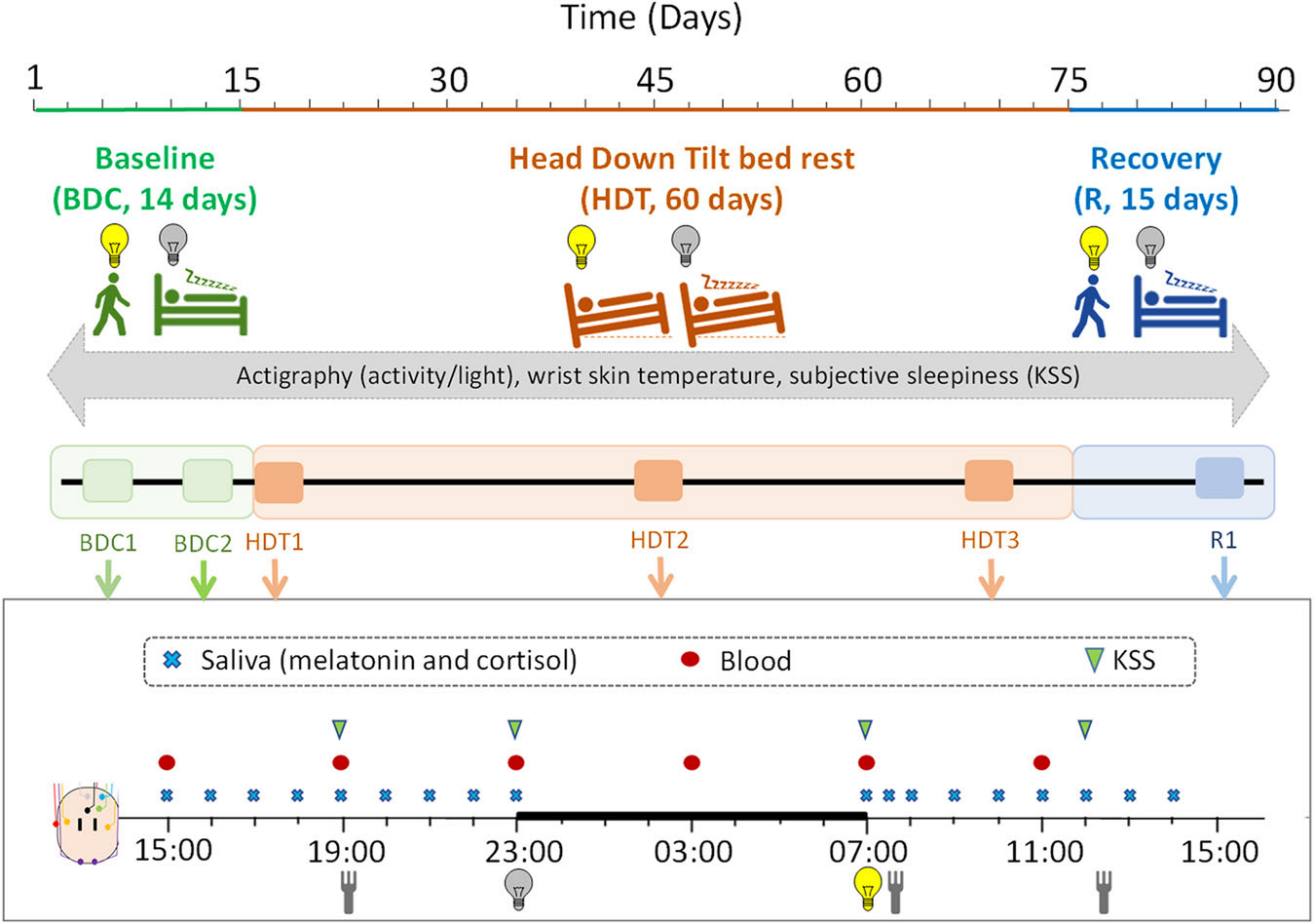
Protocol of the study. Each campaign was divided in three segments: Baseline Data Collection (BDC, 14 days), Head Down Tilt bed-rest (HDT, 60 days) and Recovery (R, 14 days). Yellow and grey bulbs indicate lights on and lights off, respectively; forks indicate mealtimes; green triangles indicate Karolinska Sleepiness Scale (KSS) test; red circles indicate blood sampling (for transcriptome analysis, not presented); blue crosses indicate saliva sampling (for melatonin and cortisol detection).

The study was conducted at MEDES (Institute for Space Medicine and Physiology, Toulouse, France) and was divided in two campaigns, the first occurring January-April 2017, and the second in September-December 2017. Each of these campaigns enrolled 10 volunteers and consisted of three segments. (1) 14 days of baseline data collection (BDC), during which the participants were already confined to the clinic but under a normal posture schedule. (2) 60 days of head down tilt bed rest (HDT) during which the participants were in bed 24 h/day with 6 degrees head down tilt. (3) 14 days of recovery (R), with identical conditions as for BDC (Fig. 8). During the whole study, the sleep periods were scheduled from 23:00 (lights off) to 07:00 (lights on). Breakfast was served at around 7:30, lunch around 12:30 and dinner around 19:30 (Fig. 8). The volunteers also ate an afternoon snack around 16:00. During HDT, the participants performed all hygiene and toilet tasks in bed, always keeping at least one shoulder in contact with bed. Showers were performed in cradles with 6 degrees head down tilt.

The participants entered the study staggered by pairs, the first pair entering on the first day, the second pair on the second day, etc. Thus, the sampling was also staggered by pairs according to the specific schedule of each pair of participants. Bedrooms were double-occupied by each pair of participants and were temperature controlled (20–25 ± 0.5 °C). Participants were exposed to room lights and external light via windows throughout all segments of the protocol (see Results, section ‘Light exposure’).

For each campaign, the group was randomly divided into ‘Control’ (5 + 5 participants) and ‘Cocktail’ (5 + 5 participants) subgroups. ‘Cocktail’ participants took a combination of putative anti-inflammatory compounds named XXS 2A-BR2 (see supplementary Information). For the purpose of the current manuscript, this intervention is not considered further. We have included it as a covariate in our statistical analyses and report the effects in the supplementary material.

During the BDC period the volunteers were required to perform a protocol of treadmill or bicycle exercises for 20 to 30 min and soft stretching exercises to keep fit and avoid cardiovascular deconditioning before the HDT periods. To recover their fitness before discharge from the research facility, the subjects performed exercise for 20 to 30 min daily during the recovery period, and especially during the second week when most of the scientific assessments were performed. The exercises were adapted (walking, treadmill, bicycle, stretching) to the cardiovascular and muscular status of each participant and monitored by MEDES staff. Resident doctors and nurses attended to participants and assisted with sample collection and routine assessments.

Motor activity (MA), light exposure (L), wrist skin temperature (WST) and subjective sleepiness (Karolinska Sleepiness Scale, KSS) were continuously assessed throughout the study, while sleep and waking electroencephalography (EEG), and saliva melatonin and cortisol were sampled at intervals during specific selected 24-h episodes (blood samples were also collected for transcriptomic analyses and these data are presented elsewhere^77^) (see Fig. 8). Blood sampling included one night time sample collected during sleep which was performed with red headlights and involved awakening the participants, as shown in Supplementary Fig. 1 (right panel). This procedure was usually completed within 5 min. The 24-h sampling episodes occurred twice during BDC (BDC-12/-11 and BDC-4/-3), three times during HDT (HDT1/2, HDT26/27 and HDT53/54), and once during R (R + 10/ + 11) segment. Each sampling episode commenced at 15:00 of the first day (−12, −4, 1, 26, 53 and +10) and lasted till 15:00 of the second day (−11, −3, 2, 27, 54 and +11). The sampling sessions of BDC, HDT and R indicate the number of days before the start, or after the start, or after the end of HDT segment, respectively (Fig. 8).

### Statistical analyses

Data are expressed as mean ± standard error of the mean (SEM). Mean waveforms were calculated and averaged for each variable repeatedly assessed through 24 h.

Data were separately analysed with a General Linear Mixed model using the mixed procedure (PROC MIXED) in SAS® software (SAS Institute, Cary, NC), with the effect of protocol segment (BDC, HDT or R) or sampling session (BDC1, BDC2, HDT1, HDT2, HDT3 or R) as repeated measures. Pairwise comparisons were conducted regardless of whether the main effect was significant in the mixed model. Campaign, treatment and sampling or segment by campaign or treatment interaction were independent categorical fixed effects. Participant was a random effect. Apart from the effect of sampling session, the effect of segment was also tested for variables assessed only during those sampling sessions, by averaging them by segment (2 sampling sessions for BDC, 3 for HDT and 1 for R). The degrees of freedom were adjusted by the method of Kenward and Roger. To allow for unequal time spacing, the covariance structure of the repeated measurements is assumed to follow a spatial power (sp(pow)) structure. Dependent variables were logarithmically (base 10) transformed prior to analysis, if they did not conform to a normal distribution, following visual inspection of a plot of ranked normal-transformed model residuals. To assess the evolution of wrist skin temperature across sleep stages (N1, N2, N3, and REM) during each segment (BDC, HDT, R), we conducted an analysis of variance (ANOVA) using a general linear model (GLM) with Holm-Bonferroni correction for p-values^78^. Holm-Bonferroni adjustment was only performed on p-values for multiple comparisons. Significance level was fixed at p_adj_ < 0.05, after Holm-Bonferroni adjustment (using R software). Figures and tables include raw means and standard error of the means.

Cross-correlations were performed between wrist skin temperature vs. melatonin concentrations and sleep probability. 10-minute step lags were computed and the range from −120 to +120 min was represented. Then correlation coefficients were averaged by lag and sampling session. Positive lags indicate wrist skin temperature profile was advanced with respect to melatonin profile.

EEG spectral records with insufficient data were excluded, resulting in the sample sizes provided. Spectral power ratios with respect to baseline (calculated as the mean for BDC1 and BDC2, or just for BDC1 or BDC2 in case data for either BDC1 or BDC2 were missing) were geometrically averaged among participants, and a 95% confident interval (CI) was calculated. For that, spectral ratios with respect to BDC (or BDC1, or BDC2) were log-transformed. Then, the mean and the upper and inferior limits for 95% CI were calculated across participants. For representations, both means and 95% CI were presented in their unlogged form. Spectral data are skewed and a log to the base10 transformation usually makes them follow a normal distribution. The difference in arithmetic mean of the log transformed data is back transformed by taking the 10 to the power of the difference in arithmetic mean to compute the ratio of the geometric mean. The 95% confidence interval of the difference in arithmetic mean will also be similarly back transformed to compute the 95% confidence interval of the geometric mean. Significance was set at *p* < 0.05 testing whether log values of the ratios were different from 0.

### Actigraphy and light exposure

To assess motor activity patterns, actigraphy recordings were obtained continuously during the study (Actiwatch Spectrum, Philips Respironics, Linton Instrumentation, Palgrave Norfolk, UK). The device was worn on the non-dominant wrist and programmed to store data once every minute. The data from the actigraphs were downloaded and the batteries recharged at different points along the study and returned to the participants within less than 4 h.

We also recorded personal exposure to white light (lux) with the Actiwatch Spectrum at 1-minute epochs, as well as environmental light in the rooms, by means of an Actiwatch device attached to the wall, between the two beds. Both motor activity and light exposure data were independently analysed using SAS software (v 9.4, SAS Institute, North Carolina, USA). Data from actigraphy/light were computed every 30 min and averaged for 24 h (mean), for daytime (08:00–20:00) and night-time (00:00−07:00) and the difference was computed in order to obtain a measure of amplitude. These parameters (mean, daytime, night-time, evening and amplitude) for light exposure, motor activity and wrist skin temperature were computed by participant and segment/sampling session and included in the mixed model for statistical analysis. In order to assess the light levels participants were exposed to during saliva sampling, the evening (18:00–23:00) light (both personal and environmental) during the sampling sessions was calculated. Data were computed by participant (room in the case of environmental light) and sampling session and included in the mixed model for statistical analysis.

### Wrist Skin Temperature (WST)

The wrist skin temperature (WST) rhythm was continuously assessed across the study by means of a temperature data logger (Thermochron iButtonDS1921H, Dallas, USA) with a sensitivity of 0.125 °C and programmed to sample once every 10 min. It was attached to a double-sided cotton sport wristband, and the sensor surface was positioned on the radial artery of the non-dominant hand, as previously described^30,79^. Body temperature data were visually inspected, and artefacts were removed.

Data from wrist skin temperature were computed every 30 min and averaged for 24 h (mean), for daytime (08:00−20:00) and night-time (00:00−07:00) and the difference was computed in order to obtain a measure of amplitude. These parameters (mean, daytime, night-time and amplitude) for light exposure, motor activity and wrist skin temperature were computed by participant and segment and included in the mixed model for statistical analysis.

### Subjective sleepiness

The KSS is a sensitive and validated tool to assess sleepiness^80^ and was completed by the participants four times every day: just before breakfast (BF); just before lunch (L); just before dinner (D); and just before bed time (BT) (Fig. 8). The average score (mean) was also calculated as the mean of those four timepoints. A measure of the amplitude was also calculated as the difference between KSS score just before lunch and before bedtime. These scores (BF, L, D and BT), as well as the average score and amplitude were computed by participant and segment or sampling session and included in the mixed model.

### Saliva collection for melatonin and cortisol measurement

To measure melatonin and cortisol concentration, saliva samples were collected hourly into labelled vials during specific 24-h episodes through the protocol (as outlined above) from 7:00 to 23:00, taking an extra sample at 7:30 to capture the awakening cortisol peak. Two millilitres were taken and frozen at −20 °C immediately after collection.

Melatonin concentrations were quantified by radioimmunoassay (Stockgrand Ltd., University of Surrey, Guildford, UK), with a detection limit of 0.85 pg mL per mL. The intra assay coefficients of variation (CV) for the low (mean ± SD, 6.3 ± 1.0 pg per mL), medium (29.3 ± 3.7 pg per mL) and high (58.7 ± 6.3 pg per mL) pools were 15.9, 12.5 and 10.7%, respectively. All samples collected for each participant were measured in duplicate in a single assay.

For analyses, melatonin concentration data were computed by participant and sampling session including all time points (07:00–12:00 and 18:00–23:00, mean) or only morning (07:00–12:00) or evening (18:00–23:00) concentrations, and these data were included in the mixed model. The phase marker melatonin onset (MOn) was calculated as described by Voultsios et al. ^81^, i.e., determining a threshold from the mean plus two standard deviations of three baseline samples. For melatonin offset (MOff), the same threshold was used, but the calculation for MOff considered the time when melatonin was below that threshold. For statistical analysis, MOn and MOff were computed by participant and sampling session and then entered in the mixed model.

Cortisol concentrations were also quantified by radioimmunoassay (Stockgrand Ltd., University of Surrey, Guildford, UK), with a detection limit of 0.7 nmol per L. The intra assay coefficients of variation (CV) for the low (mean ± SD, 2.7 ± 0.3 nmol per L), medium (16.0 ± 1.6 nmol per L) and high (46.3 ± 5.5 nmol per L) pools were 13.0, 10.2 and 12.0%, respectively. All samples collected for each participant were measured in duplicate in a single assay. For statistical analysis, cortisol concentration data were computed by participant and sampling session including all time points (mean) or considering only morning (07:00–14:00) or evening concentrations (15:00–23:00).

Cortisol peak time was computed as the time when cortisol concentration was maximum, per participant and sampling session.

### Electroencephalography (EEG) recordings

Twenty-four-hour EEG was recorded during the six 24-h sessions in BDC, HDT, and R using a portable 6-channel EEG device (SOMNOtouch ^TM^ RESP, SOMNOmedics GmbH Randersacker Germany). One mastoid reference electrode, one forehead ground electrode, two EOG electrodes, three EEG electrodes (O1, C3, F3), and two EMG electrodes were located using the international 10–20 system and mounted with Grass EC2 electrode cream (Natus Medical Inc., United States). All sensors were connected to the headbox attached to the person with a belt around the waist and connected to the EEG device. Data were sampled at 128 Hz and internally recorded and stored in the device. EEG recording started at 15:00 day 1 and ended at 15:00 day 2 of each sampling session.

The data files were randomised for blinding before being read into the software DOMINO^®^ (SOMNOmedics Gmbh, Germany) for visualisation and scoring. Each dataset was manually scored following the guidelines of the American Academy of Sleep Science (AASM) to obtain the hypnograms. Sleep probability throughout the 24-h recording period was calculated by dichotomising as “asleep” equal to 1 and “awake” equal to 0. Then, mean waveforms were calculated by computing data over 10-minute periods. Then these values were averaged among all participants.

Sleep parameters (nocturnal sleep duration from 23:00 to 07:00, diurnal sleep duration from 07:00 to 23:00, sleep efficiency, REM latency and percentage spent in N3 and REM during nocturnal sleep) were extracted from these scores using SAS (SAS Institute, Cary, NC). These parameters, computed by participant and sampling session, were included in the mixed model.

EEG power spectrum analysis was conducted after artefact removal by visual inspection of all records, for all nights that had sufficient data available. EEG power spectra were computed on 4 s epochs with 25% overlap between consecutive epochs; for each 30 s epoch an average power spectrum was computed (see^82^ for description of spectral analysis procedures). For each 1 Hz bin, between 1 and 32 Hz, the mean of the 0.25 Hz data was calculated (e.g., for 1 Hz, the mean of 0.25, 0.50, 0.75 and 1 Hz was computed). Data from the C3-M2 channel were used.

### Reporting summary

Further information on research design is available in the Nature Research Reporting Summary linked to this article.

### Supplementary information

Reporting Summary

supplementary Material

## Data Availability

Datasets and SAS code available on request.
