# Author Correction: Effect of 60 days of head down tilt bed rest on amplitude and phase of rhythms in physiology and sleep in men

**DOI:** 10.1038/s41526-024-00394-4

**Published:** 2024-04-23

**Authors:** María-Ángeles Bonmatí-Carrión, Nayantara Santhi, Giuseppe Atzori, Jeewaka Mendis, Sylwia Kaduk, Derk-Jan Dijk, Simon N. Archer

**Affiliations:** 1 Surrey Sleep Research Centre, Faculty of Health and Medical Sciences, University of Surrey, Guildford, UK; 2 Surrey Clinical Trials Unit, Faculty of Health and Medical Sciences, University of Surrey, Guildford, UK; 3 UK Dementia Research Institute Care Research and Technology Centre, Imperial College London and the University of Surrey, Guildford, UK; 4 Present Address: Chronobiology Laboratory, Department of Physiology, IMIB-Arrixaca, University of Murcia, Murcia, Spain; 5 Present Address: CIBER de Fragilidad y Envejecimiento Saludable, Instituto de Salud Carlos III, Madrid, Spain; 6 Present Address: Department of Psychology, Northumbria University, Newcastle Upon Tyne, UK

**Keywords:** Physiology, Neuroscience

Correction to: *npj Microgravity* 10.1038/s41526-024-00387-3, published online 29 March 2024

In the “Acknowledgements” section, the funding from the European Space Agency (ESA) for the bed rest facility was omitted. The original article has been corrected: ‘The bed rest facility funding was provided by the European Space Agency (ESA).

