# Supplementary material for: Effect of 60 days of head down tilt bed rest on amplitude and phase of rhythms in physiology and sleep in men
Source: NPJ Microgravity. 2024 Mar 29;10:42. doi: 10.1038/s41526-024-00387-3 (PMC10980770; doi:10.1038/s41526-024-00387-3)
Supplement: Supplementary file 1 — Reporting Summary [file 41526_2024_387_MOESM1_ESM.pdf]

nature portfolio

Corresponding author(s): Simon N. Archer

Last updated by author(s): Mar 4, 2024

Reporting Summary

Nature Portfolio wishes to improve the reproducibility of the work that we publish. This form provides structure for consistency and transparency in reporting. For further information on Nature Portfolio policies, see our Editorial Policies and the Editorial Policy Checklist.

Statistics

For all statistical analyses, confirm that the following items are present in the figure legend, table legend, main text, or Methods section.

n/a	Confirmed	
☐	☒	The exact sample size (n) for each experimental group/condition, given as a discrete number and unit of measurement
☐	☒	A statement on whether measurements were taken from distinct samples or whether the same sample was measured repeatedly
☐	☒	The statistical test(s) used AND whether they are one- or two-sided<br>*Only common tests should be described solely by name; describe more complex techniques in the Methods section.*
☐	☒	A description of all covariates tested
☐	☒	A description of any assumptions or corrections, such as tests of normality and adjustment for multiple comparisons
☐	☒	A full description of the statistical parameters including central tendency (e.g. means) or other basic estimates (e.g. regression coefficient) AND variation (e.g. standard deviation) or associated estimates of uncertainty (e.g. confidence intervals)
☐	☒	For null hypothesis testing, the test statistic (e.g. F, t, r) with confidence intervals, effect sizes, degrees of freedom and P value noted<br>*Give P values as exact values whenever suitable.*
☒	☐	For Bayesian analysis, information on the choice of priors and Markov chain Monte Carlo settings
☐	☒	For hierarchical and complex designs, identification of the appropriate level for tests and full reporting of outcomes
☒	☐	Estimates of effect sizes (e.g. Cohen's d, Pearson's r), indicating how they were calculated

Our web collection on statistics for biologists contains articles on many of the points above.

Software and code

Policy information about availability of computer code

Data collection	For motor activity and light exposure: Actiwatch Spectrum, Philips Respironics, Linton Instrumentation, Palgrave Norfolk, UK.<br>For wrist skin temperature: iButton data logger, Thermochron iButtonDS1921H, Dallas, USA.<br>For polysomnography: 6-channel EEG device, SOMNOtouch TM RESP, SOMNOmedics, S-Med, Redditch, UK.
Data analysis	SAS® software (v 9.4); RStudio 2023.3.1

For manuscripts utilizing custom algorithms or software that are central to the research but not yet described in published literature, software must be made available to editors and reviewers. We strongly encourage code deposition in a community repository (e.g. GitHub). See the Nature Portfolio guidelines for submitting code & software for further information.

Data

Policy information about availability of data

All manuscripts must include a data availability statement. This statement should provide the following information, where applicable:

- Accession codes, unique identifiers, or web links for publicly available datasets
- A description of any restrictions on data availability
- For clinical datasets or third party data, please ensure that the statement adheres to our policy

Datasets and SAS code available on request.

Research involving human participants, their data, or biological material

Policy information about studies with human participants or human data. See also policy information about sex, gender (identity/presentation), and sexual orientation and race, ethnicity and racism.

Reporting on sex and gender	All participants included in this study were men. No information about their gender identity or sexual orientation was obtained.
Reporting on race, ethnicity, or other socially relevant groupings	Black African origin (1)<br>African Caribbean origin (1)<br>Latin American origin (1)<br>White (17)
Population characteristics	Twenty healthy non-smokers male volunteers (age 20-45, 34.15 ± 7.63, mean ± SD)
Recruitment	The call for candidates was made via Internet on the MEDES and ESA sites and by media. The candidates had to fulfill an application file and sent it to MEDES. Then, MEDES contacted them to provide them with more information about the protocol and eventually to obtain clarification on specific issue. The selection was carried out in three phases:<br>- One preliminary selection on the base of the application files, comprising first a questionnaire on the subject's way of life, his education and professional experience; and second a medical questionnaire on the personal and family medical history.<br>- A second phase for complete information of the volunteers during a dedicated meeting. The investigators described precisely the whole study to the volunteers and answered to all the questions. The goal of this information session was to clarify everything with the applicants and to give them a reflection period before the next selection step.<br>- A selection session, comprising clinical and paramedical examinations, carried out where the investigations was undertaken (Medes Space Clinic).<br>The pre-selected volunteers received an information and consent document, so that they were fully informed and in a position to make their decision to participate or not in the experiment (after having carefully read the information document).<br><br>These volunteers were then notified of a selection visit at MEDES to check if they do (or do not) meet the requirements of the criteria listed below:<br><br>Subject Aptitude:<br>Candidates were declared apt to participate in the experiment by the following conditions:<br>After verification that the information document has been read carefully, candidates had the possibility to ask any additional question concerning the research project. After having obtained satisfactory answers to these questions, they had to sign the specific Information and Consent Form. After checking the results of the tests, verification was made that all inclusion criteria are present and that there are no non-inclusion criteria.
Ethics oversight	Comité de Protection des Personnes SUD-OUEST ET OUTRE-MER I, and by the Agence nationale de sécurité du medicament et des produits de santé

Note that full information on the approval of the study protocol must also be provided in the manuscript.

Field-specific reporting

Please select the one below that is the best fit for your research. If you are not sure, read the appropriate sections before making your selection.

☒ Life sciences ☐ Behavioural & social sciences ☐ Ecological, evolutionary & environmental sciences

For a reference copy of the document with all sections, see nature.com/documents/nr-reporting-summary-flat.pdf

Life sciences study design

All studies must disclose on these points even when the disclosure is negative.

Sample size	A sample size calculation was not possible with current knowledge. Considering the previous bedrest studies results and assuming a certain dropout rate a sample size of n=20 should suffice to obtain significant results.
Data exclusions	For spectral analyses, 2-3 measurements were excluded for bad quality of the signal to perform this analysis.
Replication	Not sure if this applies.
Randomization	Each volunteer was randomly allocated by drawing lots to one of the two compared groups (Control or Cocktail group) the week before the beginning of the study when the two roommates were assigned in each bedroom.
Blinding	Investigators were blinded in terms of group allocation (cocktail treatment or not). For EEG analyses, researcher were also blinded to condition, group and campaign. However, during data collection, because of the head down tilt bed rest condition nature itself, investigators could not be blinded to that or to campaign.

Reporting for specific materials, systems and methods

We require information from authors about some types of materials, experimental systems and methods used in many studies. Here, indicate whether each material, system or method listed is relevant to your study. If you are not sure if a list item applies to your research, read the appropriate section before selecting a response.

Materials & experimental systems

n/a	Involved in the study	
☐	☒	Antibodies
☒	☐	Eukaryotic cell lines
☒	☐	Palaeontology and archaeology
☒	☐	Animals and other organisms
☒	☐	Clinical data
☒	☐	Dual use research of concern
☒	☐	Plants

Methods

n/a	Involved in the study	
☒	☐	ChIP-seq
☒	☐	Flow cytometry
☒	☐	MRI-based neuroimaging

Antibodies

Antibodies used	Included in radioimmunoassays for cortisol and melatonin quantification (Stockgrand Ltd., University of Surrey, Guildford, UK).
Validation	*Describe the validation of each primary antibody for the species and application, noting any validation statements on the manufacturer's website, relevant citations, antibody profiles in online databases, or data provided in the manuscript.*

Plants

Seed stocks	*Report on the source of all seed stocks or other plant material used. If applicable, state the seed stock centre and catalogue number. If plant specimens were collected from the field, describe the collection location, date and sampling procedures.*
Novel plant genotypes	*Describe the methods by which all novel plant genotypes were produced. This includes those generated by transgenic approaches, gene editing, chemical/radiation-based mutagenesis and hybridization. For transgenic lines, describe the transformation method, the number of independent lines analyzed and the generation upon which experiments were performed. For gene-edited lines, describe the editor used, the endogenous sequence targeted for editing, the targeting guide RNA sequence (if applicable) and how the editor was applied.*
Authentication	*Describe any authentication procedures for each seed stock used or novel genotype generated. Describe any experiments used to assess the effect of a mutation and, where applicable, how potential secondary effects (e.g. second site T-DNA insertions, mosiacism, off-target gene editing) were examined.*
