# Supplementary material for: Effect of 60 days of head down tilt bed rest on amplitude and phase of rhythms in physiology and sleep in men
Source: NPJ Microgravity. 2024 Mar 29;10:42. doi: 10.1038/s41526-024-00387-3 (PMC10980770; doi:10.1038/s41526-024-00387-3)
Supplement: Supplementary file 2 — supplementary Material [file 41526_2024_387_MOESM2_ESM.pdf]

Effect of 60 days of head down tilt bed rest on amplitude and phase of rhythms in physiology and sleep in men

María-Ángeles Bonmatí-Carrión*[1,4], Nayantara Santhi[1,5], Giuseppe Atzori[1], Jeewaka Mendis[2], Sylwia Kaduk[1], Derk-Jan Dijk[1,3], Simon N. Archer*[1].

[1]Surrey Sleep Research Centre, Faculty of Health and Medical Sciences, University of Surrey, Guildford, UK

[2]Surrey Clinical Trials Unit, Faculty of Health and Medical Sciences, University of Surrey, Guildford, UK

[3]UK Dementia Research Institute Care Research and Technology Centre, Imperial College London and the University of Surrey, Guildford UK

[4]Current affiliation: Chronobiology Laboratory, Department of Physiology, IMIB-Arrixaca, University of Murcia, Murcia, Spain. CIBER de Fragilidad y Envejecimiento Saludable, Instituto de Salud Carlos III.

[5]Current affiliation: Department of Psychology, Northumbria University, Newcastle Upon Tyne, UK

*Corresponding authors: simon.archer@surrey.ac.uk, mabonmati@um.es

Key words: sleep quality, microgravity, entrainment, melatonin, cortisol, wrist skin temperature, EEG

Supplementary information

Supplementary Tables

Supplementary Table1. Type 3 Tests of Fixed effects for variables measured across the study. Factors: campaign, group, segment, and interactions.

	Statistics	Campaign (seasonal effect)	Group	Segment	Group*Segment	Campaign*Segment
Personal Light Exposure (Mean)	Pr > F<br>Num DF<br>Den DF<br>F Value	0.1671<br>1<br>17<br>2.08	0.6769<br>1<br>17<br>0.18	0.0007*<br>2<br>34<br>9.09	0.0562<br>2<br>34<br>3.14	0.0010*<br>2<br>34<br>8.50
Personal Light Exposure (daytime)	Pr > F<br>Num DF<br>Den DF<br>F Value	0.1622<br>1<br>17<br>2.14	0.6769<br>1<br>17<br>0.18	0.0015*<br>2<br>34<br>7.96	0.0549<br>2<br>34<br>0.84	0.0011*<br>2<br>34<br>3.26
Personal Light Exposure (night-time)	Pr > F<br>Num DF<br>Den DF<br>F Value	0.8716<br>1<br>17<br>0.03	0.051<br>1<br>17<br>4.38	0.1268<br>2<br>34<br>2.20	0.6709<br>2<br>34<br>0.40	0.0.5998<br>2<br>34<br>0.52
Personal Light Exposure (amplitude)	Pr > F<br>Num DF<br>Den DF<br>F Value	0.1631<br>1<br>17<br>2.13	0.4497<br>1<br>17<br>0.48	0.0022*<br>2<br>34<br>7.38	0.0402*<br>2<br>34<br>3.54	0.0003*<br>2<br>34<br>10.68
Motor Activity (Mean)	Pr > F<br>Num DF<br>Den DF<br>F Value	0.6041<br>1<br>17<br>0.28	0.6353<br>2<br>34<br>0.82	<.0001*<br>2<br>34<br>145.45	0.4488<br>2<br>34<br>0.82	0.1784<br>2<br>34<br>1.81
Motor Activity (daytime)	Pr > F<br>Num DF<br>Den DF<br>F Value	0.8953<br>1<br>17<br>0.02	0.8958<br>1<br>17<br>0.02	<.0001*<br>2<br>34<br>186.37	0.4767<br>2<br>34<br>0.76	0.3935<br>2<br>34<br>0.96
Motor Activity (night-time)	Pr > F<br>Num DF<br>Den DF<br>F Value	0.0465*<br>1<br>17<br>4.61	0.3751<br>1<br>17<br>0.83	0.1806<br>2<br>34<br>1.80	0.6519<br>2<br>34<br>0.43	0.0146*<br>2<br>34<br>4.80
Motor Activity (amplitude)	Pr > F<br>Num DF<br>Den DF<br>F Value	0.7715<br>1<br>17<br>0.09	0.8470<br>1<br>17<br>0.04	<.0001*<br>2<br>34<br>167.88	0.5111<br>2<br>34<br>0.68	0.3852<br>2<br>34<br>0.98
Wrist Skin Temperature (Mean)	Pr > F<br>Num DF<br>Den DF<br>F Value	0.0475*<br>1<br>17<br>4.56	0.4475<br>1<br>17<br>0.60	<.0001*<br>2<br>34<br>12.62	0.5905<br>2<br>34<br>0.54	0.0022*<br>2<br>34<br>7.39
Wrist Skin Temperature (night-time)	Pr > F<br>Num DF<br>Den DF<br>F Value	0.6254<br>1<br>17 0.25	0.6905<br>1<br>17<br>0.16	0.0683<br>2<br>34<br>2.91	0.5114<br>2<br>34<br>0.68	0.3979<br>2<br>34<br>0.95
Wrist Skin Temperature (daytime)	Pr > F<br>Num DF<br>Den DF<br>F Value	0.0659<br>1<br>17<br>3.86	0.2764<br>1<br>17<br>1.26	<.0001*<br>2 34<br>33.10	0.3079<br>2<br>34<br>1.22	<.0001*<br>2<br>34<br>14.74

Wrist Skin Temperature (amplitude)	Pr > F<br>Num DF<br>Den DF<br>F Value	0.3978<br>1<br>17<br>0.75	0.3212<br>1<br>17<br>1.04	<.0001*<br>2<br>34<br>23.24	0.1729<br>2<br>34<br>1.85	0.0001*<br>2<br>34<br>11.95
Subjective Sleepiness (mean)	Pr > F<br>Num DF<br>Den DF<br>F Value	0.1272<br>1<br>17<br>2.57	0.4083<br>1<br>17<br>0.72	0.1341<br>2<br>34<br>2.13	0.6746<br>2<br>34<br>0.40	0.8727<br>2<br>34<br>0.14
Subjective Sleepiness (before breakfast)	Pr > F<br>Num DF<br>Den DF<br>F Value	0.0057*<br>1<br>17<br>9.98	0.0694<br>1<br>17<br>3.76	0.2149<br>2<br>34<br>1.61	0.6141<br>2<br>34<br>0.49	0.7427<br>2<br>34<br>0.30
Subjective Sleepiness (before lunch)	Pr > F<br>Num DF<br>Den DF<br>F Value	0.1691<br>1<br>17<br>2.06	0.0673<br>1<br>17<br>3.82	0.0016*<br>2<br>34<br>7.85	0.6812<br>2<br>34<br>0.39	0.9508<br>2<br>34<br>0.05
Subjective Sleepiness (before dinner)	Pr > F<br>Num DF<br>Den DF<br>F Value	0.2383<br>1<br>17<br>1.49	0.2041<br>1<br>17<br>1.74	0.0432*<br>2<br>34<br>3.45	0.2897<br>2<br>34<br>1.29	0.1219<br>2<br>34<br>2.24
Subjective Sleepiness (before bed time)	Pr > F<br>Num DF<br>Den DF<br>F Value	0.8495<br>1<br>17<br>0.04	0.4035<br>1<br>17<br>0.73	0.0899<br>2<br>34<br>2.59	0.1615<br>2<br>34<br>1.92	0.1064<br>2<br>34<br>2.40
Subjective Sleepiness (amplitude)	Pr > F<br>Num DF<br>Den DF<br>F Value	0.7007<br>1<br>17<br>0.15	0.1241<br>1<br>17<br>2.62	0.0002*<br>2<br>34<br>10.85	0.0830<br>2<br>34<br>2.68	0.0718<br>2<br>34<br>2.85

Campaign is referred to the leg the study was split into (January-April or September-December). Group refers to the cocktail treatment group (results detailed below in this supplementary material). Segment is the phase of the study: baseline data collection (BDC), head-down tilt (HDT) or recovery (R). Num DF: numerator degrees of freedom; Den DF: denominator degrees of freedom.

Supplementary Table 2. Differences of least squares means for segment on variables measured across the study.

		BDC vs HDT	BDC vs R	HDT vs R
Personal Light Exposure (Mean)	Pr > |t| Holm-adj. p-value<br>Estimate<br>Standard Error<br>DF<br>t Value	0.0002*<br>0.0006*<br>-13.8206<br>3.3741<br>34<br>-4.10	0.3127<br>0.3127<br>-3.4578<br>3.3741<br>34<br>-1.02	0.0042*<br>0.0084*<br>-3.4578<br>3.3741<br>34<br>-3.07
Personal Light Exposure (daytime)	Pr > |t| Holm-adj. p-value<br>Estimate<br>Standard Error<br>DF<br>t Value	0.0010*<br>0.003*<br>-18.3053<br>5.0979<br>34<br>-3.59	0.7761<br>0.7761<br>-1.4615<br>5.0979<br>34<br>-0.29	0.0023*<br>0.0046*<br>-16.8438<br>5.0979<br>34<br>-3.30
Personal Light Exposure (night-time)	Pr > |t| Holm-adj. p-value<br>Estimate<br>Standard Error	0.0510<br>0.153<br>-2.7365<br>1.3526	0.1469<br>0.2938<br>-2.0081<br>1.3526	0.5937<br>0.5937<br>-0.7284<br>1.3526

	DF	34	34	34
	t Value	-2.02	-1.48	-0.54
Personal Light Exposure (amplitude)	Pr > |t|	0.0025*	0.9093	0.0018*
	Holm-adj. p-value	0.0054*	0.9093	0.0054*
	Estimate	-15.5688	0.5466	-16.1154
	Standard Error	4.7634	4.7634	4.7634
	DF	34	34	34
	t Value	-3.27	0.11	-3.38
Motor Activity (Mean)	Pr > |t|	<.0001*	0.1934	<.0001*
	Holm-adj. p-value	0.0003*	0.1934	0.0003*
	Estimate	-50.1236	-4.3214	-45.8021
	Standard Error	3.2571	3.2571	3.2571
	DF	34	34	34
	t Value	-15.39	-1.33	-14.06
Motor Activity (daytime)	Pr > |t|	<.0001*	0.0705	<.0001*
	Holm-adj. p-value	0.0003*	0.0705	0.0003*
	Estimate	-0.2506	-0.02662	-0.2240
	Standard Error	0.01426	0.01426	0.01426
	DF	34	34	34
	t Value	-17.58	-1.87	-15.71
Motor Activity (night-time)	Pr > |t|	0.7849	0.0868	0.1459
	Holm-adj. p-value	0.7849	0.2604	0.2918
	Estimate	-0.00596	-0.03819	0.03223
	Standard Error	0.02166	0.02166	0.02166
	DF	34	34	34
	t Value	-0.28	-1.76	1.49
Motor Activity (amplitude)	Pr > |t|	<.0001*	0.0439*	<.0001*
	Holm-adj. p-value	0.0003*	0.0439*	0.0003*
	Estimate	-86.0236	-10.7108	-75.3128
	Standard Error	5.1169	5.1169	5.1169
	DF	34	34	34
	t Value	-16.81	-2.09	-14.72
Wrist Skin Temperature (Mean)	Pr > |t|	<.0001*	0.0866	0.0030*
	Holm-adj. p-value	0.0003*	0.0866	0.006*
	Estimate	0.2765	0.09844	0.1780
	Standard Error	0.05579	0.05579	0.05579
	DF	34	34	34
	t Value	4.96	1.76	3.19
Wrist Skin Temperature (night-time)	Pr > |t|	0.9625	0.0422*	0.0467*
	Holm-adj. p-value	0.9625	0.1266	0.1266
	Estimate	0.004003	0.1785	-0.1745
	Standard Error	0.08453	0.08453	0.08453
	DF	34	34	34
	t Value	0.05	2.11	-2.06
Wrist Skin Temperature (daytime)	Pr > |t|	<.0001*	0.5932	<.0001*
	Holm-adj. p-value	0.0003*	0.5932	0.0003*
	Estimate	0.4638	0.03426	0.4296
	Standard Error	0.06353	0.06353	0.06353
	DF	34	34	34
	t Value	7.30	0.54	6.76
Wrist Skin Temperature (amplitude)	Pr > |t|	<.0001*	0.1284	<.0001*
	Holm-adj. p-value	0.0003*	0.1284	0.0003*
	Estimate	-0.4598	0.1442	-0.6040
	Standard Error	0.09254	0.09254	0.09254
	DF	34	34	34
	t Value	-4.97	1.56	-6.53

Subjective Sleepiness (mean)	Pr > |t|	0.9552	0.0875	0.0782
	Holm-adj. p-value	0.9552	0.2346	0.2346
	Estimate	-0.00737	0.2294	-0.2368
	Standard Error	0.1304	0.1304	0.1304
	DF	34	34	34
	t Value	-0.06	1.76	-1.82
Subjective Sleepiness (before breakfast)	Pr > |t|	0.1570	0.1098	0.8467
	Holm-adj. p-value	0.3294	0.3294	0.8467
	Estimate	0.3059	0.3471	-0.04118
	Standard Error	0.2114	0.2114	0.2114
	DF	34	34	34
	t Value	1.45	1.64	-0.19
Subjective Sleepiness (before lunch)	Pr > |t|	0.0481*	0.0004*	0.0645
	Holm-adj. p-value	0.0962	0.0012*	0.0962
	Estimate	0.2875	0.5555	-0.2680
	Standard Error	0.1403	0.1403	0.1403
	DF	34	34	34
	t Value	2.05	3.96	-1.91
Subjective Sleepiness (before dinner)	Pr > |t|	0.0524	0.6486	0.0187*
	Holm-adj. p-value	0.1048	0.6486	0.0561
	Estimate	-0.3160	0.07230	-0.3883
	Standard Error	0.1572	0.1572	0.1572
	DF	34	34	34
	t Value	-2.01	0.46	-2.47
Subjective Sleepiness (before bed time)	Pr > |t|	0.0397*	0.6931	0.0907
	Holm-adj. p-value	0.1191	0.6931	0.1814
	Estimate	-0.3069	-0.05710	-0.2498
	Standard Error	0.1435	0.1435	0.1435
	DF	34	34	34
	t Value	-2.14	-0.40	-1.74
Subjective Sleepiness (amplitude)	Pr > |t|	0.0004*	0.0002*	0.9040
	Holm-adj. p-value	0.0008*	0.0006*	0.904
	Estimate	-0.5944	-0.6126	0.01818
	Standard Error	0.1497	0.1497	0.1497
	DF	34	34	34
	t Value	-3.97	-4.09	0.12

BDC: baseline data collection; HDT: head-down tilt; R: recovery. Pr > |t|: nominal p-value; Holm-adj. p-value: p-value adjusted by Holm-Bonferroni correction. DF: degrees of freedom.

Supplementary Table 3. Type 3 Tests of Fixed effects for variables measured during specific sampling sessions. Factors: campaign, group, sampling session, and interactions.

		Campaign	Group	Sampling session	Group* Sampling session	Campaign* Sampling session
Evening environmental light (18:00 – 23:00)	Pr > F	0.9178	0.8375	0.0451	0.6269	0.3618
	Num DF	1	1	4	4	4
	Den DF	17	17	85	85	85
	F Value	0.01	0.04	2.55	0.65	1.10
Evening light exposure (18:00 – 23:00)	Pr > F	0.7386	0.0310	0.1455	0.0548	0.2151
	Num DF	1	1	5	5	5
	Den DF	102	102	102	102	102
	F Value	0.11	4.78	1.68	2.25	1.44
Melatonin onset	Pr > F	0.6090	0.9621	0.1114	0.6917	0.0053*
	Num DF					

	Den DF<br>F Value	1<br>16.9<br>0.27	1<br>16.9<br>0.00	5<br>80.5<br>1.86	5<br>80.5<br>0.61	5<br>80.5<br>3.62
Melatonin offset	Pr > F<br>Num DF<br>Den DF<br>F Value	0.3655<br>1<br>17<br>0.86	0.7886<br>1<br>17<br>0.07	0.7980<br>5<br>85<br>0.47	0.5320<br>5<br>85<br>0.83	0.9808<br>5<br>85<br>0.15
Mel. conc. (mean)<br>7-12	Pr > F<br>Num DF<br>Den DF<br>F Value	0.0007*<br>1<br>17<br>16.86	0.0223*<br>1<br>17<br>6.32	0.0695<br>5<br>60.9<br>2.17	0.1303<br>5<br>60.9<br>1.78	0.4327<br>5<br>60.9<br>0.99
Mel. conc. (morning)	Pr > F<br>Num DF<br>Den DF<br>F Value	0.0009*<br>1<br>17<br>16.21	0.0473*<br>1<br>17<br>4.57	0.5678<br>5<br>64.5<br>0.78	0.3420<br>5<br>64.5<br>1.15	0.3084<br>5<br>64.5<br>1.22
Mel. conc. (evening)	Pr > F<br>Num DF<br>Den DF<br>F Value	0.0028*<br>1<br>17<br>12.22	0.0429*<br>1<br>17<br>4.79	0.0074*<br>5<br>68.6<br>3.48	0.1948<br>5<br>68.6<br>1.52	0.8689<br>5<br>68.6<br>0.37
Cortisol peak time	Pr > F<br>Num DF<br>Den DF<br>F Value	0.8741<br>1<br>102<br>0.03	0.6347<br>1<br>102<br>0.23	0.3549<br>5<br>102<br>1.12	0.6303<br>5<br>102<br>0.69	0.7191<br>5<br>102<br>0.57
Cortisol conc. (mean)	Pr > F<br>Num DF<br>Den DF<br>F Value	0.7388<br>1<br>16.7<br>0.11	0.0025*<br>1<br>16.7<br>12.56	0.0694<br>5<br>65.7<br>2.16	0.8329<br>5<br>65.7<br>0.42	0.3012<br>5<br>65.7<br>1.24
Cortisol conc.<br>(morning)	Pr > F<br>Num DF<br>Den DF<br>F Value	0.7630<br>1<br>16.7<br>0.09	0.0038*<br>1<br>16.7<br>11.24	0.0277*<br>5<br>67.5<br>2.70	0.9020<br>5<br>67.5<br>0.32	0.2136<br>5<br>67.5<br>1.46
Cortisol conc.<br>(evening)	Pr > F<br>Num DF<br>Den DF<br>F Value	0.5037<br>1<br>17.2<br>0.47	0.0087*<br>1<br>17.2<br>8.77	0.0610<br>5<br>66.6<br>2.23	0.9484<br>5<br>66.6<br>0.23	0.3378<br>5<br>66.6<br>1.16
Nocturnal sleep<br>duration	Pr > F<br>Num DF<br>Den DF<br>F Value	0.0326*<br>1<br>12.5<br>5.77	0.5012<br>1<br>12.5<br>0.48	0.6411<br>5<br>45.4<br>0.68	0.0637<br>5<br>45.4<br>2.27	0.2810<br>5<br>45.4<br>1.30
Sleep efficiency	Pr > F<br>Num DF<br>Den DF<br>F Value	0.0326*<br>1<br>12.5<br>5.77	0.5011<br>1<br>12.5<br>0.48	0.6413<br>5<br>45.4<br>0.68	0.0637<br>5<br>45.4<br>2.27	0.2813<br>5<br>45.4<br>1.30
REM latency	Pr > F<br>Num DF<br>Den DF<br>F Value	0.3613<br>1<br>16.8<br>0.88	0.8958<br>1<br>16.8<br>0.02	0.9093<br>5<br>77.3<br>0.30	0.6992<br>5<br>77.3<br>0.60	0.7262<br>5<br>77.3<br>0.57
Diurnal sleep<br>duration	Pr > F<br>Num DF<br>Den DF<br>F Value	0.6982<br>1<br>17<br>0.16	0.9442<br>1<br>17<br>0.01	0.0084*<br>5<br>85<br>3.34	0.9885<br>5<br>85<br>0.12	0.9145<br>5<br>85<br>0.29
Wrist skin<br>temperature (mean<br>over sampling<br>sessions)	Pr > F<br>Num DF<br>Den DF<br>F Value	0.5675<br>1<br>14.4<br>0.34	0.6784<br>1<br>14.4<br>0.18	0.4283<br>5<br>78.3<br>0.99	0.4844<br>5<br>78.3<br>0.90	0.5684<br>5<br>78.3<br>0.78

Motor activity (mean over sampling sessions)	Pr > F<br>Num DF<br>Den DF<br>F Value	0.6374<br>1<br>17<br>0.23	0.8310<br>1<br>17<br>0.05	<.0001*<br>5<br>85<br>20.38	0.7811<br>5<br>85<br>20.38	0.7732<br>5<br>85<br>0.50
Light exposure (mean over sampling sessions)	Pr > F<br>Num DF<br>Den DF<br>F Value	0.3818<br>1<br>17<br>0.81	0.7485<br>1<br>17<br>0.11	0.2148<br>5<br>85<br>1.45	0.0066*<br>5<br>85<br>3.47	0.1116<br>5<br>85<br>1.85
Wrist skin temperature – amplitude over sampling sessions	Pr > F<br>Num DF<br>Den DF<br>F Value	0.2025<br>1<br>17.1<br>1.76	0.2144<br>1<br>17.1<br>1.66	0.0004*<br>5<br>66.7<br>5.27	0.7211<br>5<br>66.7<br>0.57	0.0067*<br>5<br>66.7<br>3.55
Motor activity – amplitude over sampling sessions	Pr > F<br>Num DF<br>Den DF<br>F Value	0.8415<br>1<br>17<br>0.04	0.7239<br>1<br>17<br>0.13	<.0001*<br>5<br>85<br>26.63	0.2512<br>5<br>85<br>1.35	0.3876<br>5<br>85<br>1.06
Light exposure - amplitude over sampling sessions	Pr > F<br>Num DF<br>Den DF<br>F Value	0.2760<br>1<br>17<br>1.27	0.6299<br>1<br>17<br>0.24	0.1611<br>4<br>85<br>1.68	0.0127*<br>4<br>85<br>3.39	0.0414*<br>4<br>85<br>2.60
Subjective sleepiness (mean over sampling sessions)	Pr > F<br>Num DF<br>Den DF<br>F Value	0.0996<br>1<br>16.8<br>3.04	0.1673<br>1<br>16.8<br>2.08	0.1608<br>5<br>46.2<br>1.67	0.0462*<br>5<br>46.2<br>2.47	0.2577<br>5<br>46.2<br>1.36
Subjective sleepiness (before breakfast over sampling sessions)	Pr > F<br>Num DF<br>Den DF<br>F Value	0.0307*<br>1<br>18.2<br>5.48	0.1388<br>1<br>18.2<br>2.40	0.8464<br>5<br>64.2<br>0.40	0.0517<br>5<br>64.2<br>2.34	0.3399<br>5<br>64.2<br>1.16
Subjective sleepiness (before lunch over sampling sessions)	Pr > F<br>Num DF<br>Den DF<br>F Value	0.0433*<br>1<br>17<br>4.77	0.0747<br>1<br>17<br>3.60	0.3987<br>5<br>85<br>1.04	0.7704<br>5<br>85<br>0.51	0.1723<br>5<br>85<br>1.59
Subjective sleepiness (before dinner over sampling sessions)	Pr > F<br>Num DF<br>Den DF<br>F Value	0.2880<br>1<br>17<br>1.20	0.1355<br>1<br>17<br>2.46	0.0078*<br>5<br>85<br>3.38	<.0001*<br>5<br>85<br>6.41	0.2552<br>5<br>85<br>1.34
Subjective sleepiness (before bedtime over sampling sessions)	Pr > F<br>Num DF<br>Den DF<br>F Value	0.5195<br>1<br>17<br>0.43	0.4326<br>1<br>17<br>0.65	0.6607<br>4<br>84.6<br>0.60	0.2075<br>4<br>84.6<br>1.51	0.0734<br>4<br>84.6<br>2.22
% of total sleep in N3	Pr > F<br>Num DF<br>Den DF<br>F Value	0.0171*<br>1<br>16.9<br>6.99	0.1475<br>1<br>16.9<br>2.30	0.0002*<br>5<br>59.9<br>5.92	0.2538<br>5<br>59.9<br>1.36	0.3728<br>5<br>59.9<br>1.09
% of total sleep in REM	Pr > F<br>Num DF<br>Den DF<br>F Value	0.0085*<br>1<br>16.8<br>8.89	0.2976<br>1<br>16.8<br>1.16	0.1804<br>5<br>61.4<br>1.58	0.2288<br>5<br>61.4<br>1.42	0.0872<br>5<br>61.4<br>2.03

Campaign is referred to the leg the study was split into (January-April or September-December). Group refers to the cocktail treatment group (results detailed below in this supplementary material). Sampling session refers to the specific day the data were collected: twice during baseline data collection (BDC): BDC1 (-12/-11), BDC2 (-4/-3); three times during head-down tilt bed rest (HDT): HDT1 (1/2), HDT2 (26/27) and HDT3 (53/54); and once in recovery (R): R (+10/+11). Num DF: numerator degrees of freedom; Den DF: denominator degrees of freedom.

Supplementary Table 4. Differences of least squares means for segment (BDC, HDT, R) and sampling sessions (BDC1, BDC2, HDT1, HDT2, HDT3, R).

		BDC vs HDT	BDC vs R	HDT vs R	BDC1 vs BDC2	BDC2 vs HDT1	BDC2 vs HDT3	BDC2 vs R	HDT1 vs HDT2	HDT2 vs HDT3	HDT3 vs R	BDC1 vs R	HDT1 vs R	HDT2 vs R	BDC1 vs HDT1	BDC1 vs HDT2	HDT1 v HDT3
Evening environmental light (18:00 – 23:00)	Pr > |t|	0.0764	0.0348*	0.6352	0.0697	0.8557	0.4739	0.3511	0.7637	0.9004	0.8277	0.0068*	0.5204	0.7318	0.0360*	0.0171*	0.6703
	Holm-adj. p-value	0.1528	0.1044	0.6352	0.697	1	1	1	1	1	1	0.0884	1	1	0.396	0.2052	1
	Estimate	-6.9567	-8.7932	1.8366	8.7064	1.3833	3.4061	4.4400	2.0228	0.5945	1.0339	13.1464	3.0568	1.6285	10.0897	11.5179	2.0228
	Standard Error	3.8788	4.0995	3.8570	4.7399	7.5826	4.7353	4.7353	4.7353	4.7353	4.7353	4.7353	4.7357	4.7353	4.7353	4.7353	4.7353
	DF	85.01	85	85.01	85	84.97	85	85	85	85	85	85	85	85	85	85	85
	t-Value	-1.79	-2.14	0.48	1.84	0.18	0.72	0.94	0.43	0.13	0.22	2.78	0.65	0.34	2.13	2.43	0.43
Evening light exposure (18:00 – 23:00)	Pr > |t|	0.9295	0.1428	0.1033	0.6616	0.8584	0.4310	0.2920	0.1976	0.0242*	0.7888	0.1371	0.2106	0.0120*	0.8119	0.2924	0.3240
	Holm-adj. p-value	0.9295	0.3099	0.3099	1	1	1	1	1	0.2904	1	1	1	0.156	1	1	1
	Estimate	1.3599	-29.0384	30.3983	9.9707	-4.5520	17.9531	24.0531	-29.4501	51.9552	6.0999	34.0238	28.6054	58.0552	5.4187	-24.0314	22.5051
	Standard Error	15.3257	19.6644	18.4944	22.7099	25.4476	22.703	22.7073	22.7073	22.7073	22.7073	22.7073	22.7076	22.7073	22.7073	22.7073	22.7073
	DF	102	102	102	102	102	102	102	102	102	102	102	102	102	102	102	102
	t-Value	0.09	-1.48	1.64	0.44	-0.18	0.79	1.06	-1.30	2.29	0.27	1.50	1.26	2.56	0.24	-1.06	0.99
Mel. onset	Pr > |t|	0.6932	0.1478	0.0641	0.6142	0.3524	0.6142	0.3144	0.3144	0.6142	0.1327	0.1327	0.0461*	0.3144	0.6142	0.6142	0.6142
	Holm-adj. p-value	0.6932	0.2956	0.1923	1	1	1	1	1	1	1	1	0.5993	1	1	1	1
	Estimate	-0.02600	0.1250	-0.1510	-0.05000	0.10000	0.05000	-0.10000	-0.10000	0.05000	-0.1500	-0.1500	-0.2000	-0.10000	0.05000	-0.05000	-0.05000
	Standard Error	0.06567	0.08558	0.08048	0.09882	0.1069	0.09882	0.09882	0.09882	0.09882	0.09882	0.09882	0.09882	0.09882	0.09882	0.09882	0.09882
	DF	85.01	85	85	85	85	85	85	85	85	85	85	85	85	85	85	85
	t-Value	-0.40	1.46	-1.88	-0.51	0.94	0.51	-1.01	-1.01	0.51	-1.52	-1.52	-2.02	-1.01	0.51	-0.51	-0.51
Mel. offset	Pr > |t|	0.4739	0.1274	0.2488	1.0000	1.0000	0.1765	0.1716	1.0000	0.1780	1.0000	0.1781	0.1500	0.1749	1.0000	1.0000	0.1765
	Holm-adj. p-value	0.4976	0.3822	0.4976	1	1	1	1	1	1	1	1	1	1	1	1	1
	Estimate	0.01650	0.05000	-0.03350	2.78E-17	-347E-19	-0.05000	-0.05000	-139E-19	-0.05000	-494E-19	-0.05000	-0.05000	-0.05000	-694E-20	-208E-19	-0.05000
	Standard Error	0.02293	0.03246	0.02883	0.03351	0.02967	0.03664	0.03625	0.03680	0.03679	0.03666	0.03681	0.03429	0.03655	0.03603	0.03668	0.03665
	DF	77.42	80.49	78.82	52.9	32.7	74.7	82.9	79.4	78.2	74.9	81.6	59.9	84.9	80.5	75.3	74.7
	t-Value	0.72	1.54	-1.16	0.00	-0.00	-1.36	-1.38	-0.00	-1.36	-0.00	-1.36	-1.46	-1.37	-0.00	-0.00	-1.36
Mel. conc. (mean)	Pr > |t|	0.5161	0.0713	0.0202*	0.1052	0.2346	0.0456*	0.4872	0.9728	0.4535	0.0074*	0.0165*	0.0680	0.0536	0.6297	0.6420	0.4329
	Holm-adj. p-value	0.5161	0.1426	0.0606	0.8416	1	0.5016	1	1	1	0.0962	0.198	0.612	0.536	1	1	1
	Estimate	0.01083	-0.04183	0.05266	0.04589	-0.03271	-0.05344	0.01889	-0.00090	-0.01983	0.07233	0.06477	0.05160	0.05250	0.01317	0.01227	-0.02073
	Standard Error	0.01660	0.02281	0.02225	0.02789	0.02672	0.02629	0.02706	0.02634	0.02632	0.02629	0.02640	0.02786	0.02680	0.02722	0.02629	0.02629
	DF	83.35	64.72	84.74	59.9	20.7	74.2	82.2	74.6	74.4	74.2	75.1	72.7	79.2	83.8	74.2	74.2
	t-Value	0.65	-1.83	2.37	1.65	-1.22	-2.03	0.70	-0.03	-0.75	2.75	2.45	1.85	1.96	0.48	0.47	-0.79
Mel. conc. (evening)	Pr > |t|	0.0725	0.2776	0.0129*	0.0309*	0.0015*	0.1087	0.8723	0.2684	0.5619	0.1486	0.0421*	0.0027*	0.0468*	0.2896	0.9750	0.0933
	Holm-adj. p-value	0.145	0.2776	0.0387*	0.3399	0.0195*	0.7609	1	1	1	0.8916	0.421	0.0324*	0.4212	1	1	0.7464
	Estimate	0.3045	-0.2536	0.5581	0.5942	-0.8823	-0.4310	-0.04350	0.2965	0.1548	0.3875	0.5507	0.8388	0.5423	-0.2882	0.00833	0.4513
	Standard Error	0.1674	0.2317	0.2196	0.2695	0.2539	0.2655	0.2698	0.2659	0.2658	0.2655	0.2664	0.2709	0.2686	0.2704	0.2655	0.2655
	DF	82.11	69.55	84.95	68.5	31.7	75.8	82.8	76.2	76.1	75.8	76.7	77.4	80.4	84	75.8	75.8
	t-Value	1.82	-1.09	2.54	2.20	-3.47	-1.62	-0.16	1.11	0.58	1.46	2.07	3.10	2.02	-1.07	0.03	1.70

Mel. conc. (morning)	Pr > |t|	0.9244	0.1993	0.1458	0.8707	0.8623	0.3967	0.2987	0.6439	0.5886	0.0593	0.2274	0.3690	0.1805	0.7538	0.8844	0.3153
	Holm-adj. p-value	0.9244	0.4374	0.4374	1	1	1	1	1	1	0.7709	1	1	1	1	1	1
	Estimate	0.002220	-0.04252	0.04474	0.006004	0.005887	-0.03167	0.03952	-0.01731	-0.02024	0.07119	0.04552	0.03363	0.05095	0.01189	-0.00542	-0.03755
	Standard Error	0.02332	0.03281	0.03047	0.03672	0.03363	0.03714	0.03779	0.03730	0.03725	0.03714	0.03741	0.03719	0.03772	0.03780	0.03715	0.03714
	DF	77.3	68.68	83.74	57.9	27.9	71.6	84.5	73.2	72.6	71.6	74.8	68	81.8	85	71.6	71.6
	t-Value	0.10	-1.30	1.47	0.16	0.18	-0.85	1.05	-0.46	-0.54	1.92	1.22	0.90	1.35	0.31	-0.15	-1.01
Cortisol peak time	Pr > |t|	0.9320	0.2649	0.2077	0.7166	0.6061	0.5862	0.4321	0.0924	0.1178	0.1849	0.2517	0.1484	0.8087	0.7622	0.1659	0.9037
	Holm-adj. p-value	0.9320	0.6231	0.6231	1	1	1	1	1	1	1	1	1	1	1	1	1
	Estimate	-0.02550	0.4000	-0.4255	-0.1500	0.2750	0.2250	-0.3250	-0.7000	0.6500	-0.5500	-0.4750	-0.6000	0.1000	0.1250	-0.5750	-0.05000
	Standard Error	0.2980	0.3568	0.3356	0.4121	0.5317	0.4120	0.4120	0.4120	0.4120	0.4120	0.4120	0.4120	0.4120	0.4120	0.4120	0.4120
	DF	102	102	102	102	102	102	102	102	102	102	102	102	102	102	102	102
	t-Value	-0.09	1.12	-1.27	-0.36	0.52	0.55	-0.79	-1.70	1.58	-1.33	-1.15	-1.46	0.24	0.30	-1.40	-0.12
Cortisol conc. (evening)	Pr > |t|	0.5312	0.7237	0.9200	0.1223	0.4273	0.8864	0.6874	0.0050*	0.0191*	0.5896	0.3095	0.2667	0.0646	0.0378*	0.4055	0.6176
	Holm-adj. p-value	1	1	1	1	1	1	1	0.065	0.2292	1	1	1	0.646	0.4158	1-	1
	Estimate	-0.01161	-0.00928	-0.00233	0.04209	0.01902	0.004229	-0.01177	-0.08583	0.07104	-0.01600	0.03032	-0.03079	0.05504	0.06111	0.02472	-0.01479
	Standard Error	0.01845	0.02615	0.02315	0.02683	0.02369	0.02949	0.02914	0.02964	0.02962	0.02951	0.02964	0.02748	0.02940	0.02895	0.02953	0.02949
	DF	70.16	75.73	82.34	57	35.3	67	84.7	73.6	71.9	67.4	76.9	64.4	84.6	83.3	67.9	67.1
	t-Value	-0.63	-0.35	-0.10	1.57	0.80	0.14	-0.40	-2.90	2.40	-0.54	1.02	-1.12	1.87	2.11	-0.84	-0.50
Cortisol conc. (morning)	Pr > |t|	0.0605	0.0710	0.0006*	0.1970	0.6519	0.3613	0.0277*	0.2545	0.0949	0.0024*	0.3137	0.0077	0.1519	0.1136	0.6709	0.5877
	Holm-adj. p-value	0.121	0.121	0.0018*	1	1	1	0.3047	1	0.949	0.031*	1	0.0924	1	1	1	1
	Estimate	-0.02897	0.03940	-0.06836	0.02930	0.009081	0.02231	-0.05404	-0.02801	0.04123	-0.07635	-0.02475	-0.06312	-0.03512	0.03838	0.01037	0.01323
	Standard Error	0.01520	0.02152	0.01922	0.02244	0.01996	0.02428	0.02413	0.02439	0.02438	0.02429	0.02441	0.02293	0.02429	0.02400	0.02431	0.02428
	DF	75.14	77.19	81.51	56.6	34.4	72	84.5	76.5	75.3	72.2	78.9	64.1	84.8	83.1	72.5	72
	t-Value	-1.91	1.83	-3.56	1.31	0.46	0.92	-2.24	-1.15	1.69	-3.14	-1.01	-2.75	-1.45	1.60	0.43	0.54
Cortisol conc. (mean)	Pr > |t|	0.1468	0.2303	0.0114*	0.1169	0.5521	0.6587	0.0725	0.0808	0.0832	0.0310*	0.7110	0.0163*	0.6529	0.0579	0.9433	0.9901
	Holm-adj. p-value	0.2936	0.2936	0.0342*	0.8183	1	1	0.725	0.7272	0.7272	0.372	1	0.2119	1	0.6369	1	1
	Estimate	-0.02131	0.02481	-0.04613	0.03233	0.01060	0.01031	-0.04097	-0.04126	0.04097	-0.05128	-0.00865	-0.05157	-0.01031	0.04292	0.001663	-0.00029
	Standard Error	0.01452	0.02052	0.01781	0.02029	0.01766	0.02323	0.02253	0.02331	0.02333	0.02326	0.02326	0.02087	0.02284	0.02231	0.02329	0.02324
	DF	66.78	79.16	81.09	54.3	35.7	64	83.1	74.8	72.6	64.9	78.8	60.8	85	80.7	66	64.1
	t-Value	-1.47	1.21	-2.59	1.59	0.60	0.44	-1.82	-1.77	1.76	-2.20	-0.37	-2.47	-0.45	1.92	0.07	-0.01
Subj. sleepiness (mean over sampling sessions)	Pr > |t|	0.3399	0.1116	0.4194	0.9193	0.5652	0.1526	0.1858	0.1301	0.5166	0.9720	0.2043	0.0729	0.5517	0.4237	0.5051	0.0320*
	Holm-adj. p-value	0.6798	0.3348	0.6798	1	1	1	1	1	1	1	1	0.8748	1	1	1	0.416
	Estimate	-0.1457	-0.3188	0.1731	-0.02917	-0.1750	0.3417	0.3333	0.3625	0.1542	-0.00833	0.3042	0.5083	0.1458	-0.2042	0.1583	0.5167
	Standard Error	0.1518	0.1972	0.2133	0.2863	0.2943	0.2364	0.2498	0.2368	0.2366	0.2364	0.2375	0.2791	0.2439	0.2539	0.2364	0.2364
	DF	84.61	56.88	84.57	49.6	10	72.7	79.9	72.8	72.8	72.7	73.1	68.3	76.4	82.2	72.7	72.7
	t-Value	-0.96	-1.62	0.81	-0.10	-0.59	1.45	1.33	1.53	0.65	-0.04	1.28	1.82	0.60	-0.80	0.67	2.19
Subj. sleepiness (BF over sampling sessions)	Pr > |t|	0.6491	0.2946	0.3963	0.5867	0.8749	0.8172	0.4684	0.5642	0.4899	0.6447	0.2482	0.5100	0.2362	0.5409	1.0000	0.9080
	Holm-adj. p-value	0.8838	0.8838	0.8838	1	1	1	1	1	1	1	1	1	1	1	1	1
	Estimate	-0.1230	-0.4000	0.2770	0.2000	0.05000	0.1000	0.3000	-0.2500	0.3000	0.2000	0.5000	0.2500	0.5000	0.2500	-944E-18	0.05000
	Standard Error	0.2688	0.3790	0.3249	0.3659	0.3156	0.4305	0.4119	0.4314	0.4320	0.4314	0.4296	0.3773	0.4191	0.4072	0.4319	0.4307
	DF	55.27	75.18	83.94	58.1	39.1	52.6	84.5	68.1	65.1	54.2	73.7	64.6	84.6	82.8	55.9	52.9
	t-Value	-0.46	-1.06	0.85	0.55	0.16	0.23	0.73	-0.58	0.69	0.46	1.16	0.66	1.19	0.61	-0.00	0.12

Subj. sleepiness (L over sampling sessions)	Pr > |t|	0.1837	0.6838	0.5253	0.3245	0.1357	0.4810	0.3980	0.5727	0.1605	0.8878	0.8878	0.4810	0.2061	0.5727	0.2606	0.3980
	Holm-adj. p-value	0.5511	1	1	1	1	1	1	1	1	1	1	1	1	1	1	1
	Estimate	0.3085	0.1250	0.1835	0.3500	-0.5500	-0.2500	-0.3000	-0.2000	0.5000	-0.05000	0.05000	0.2500	0.4500	-0.2000	-0.4000	0.3000
	Standard Error	0.2302	0.3059	0.2877	0.3532	0.3651	0.3532	0.3532	0.3532	0.3532	0.3532	0.3532	0.3532	0.3532	0.3532	0.3532	0.3532
	DF	85.01	85	85.01	85	85	85	85	85	85	85	85	85	85	85	85	85
	t-Value	1.34	0.41	0.64	0.99	-1.51	-0.71	-0.85	-0.57	1.42	-0.14	0.14	0.71	1.27	-0.57	-1.13	0.85
Subj. sleepiness (D over sampling sessions)	Pr > |t|	0.0085*	0.2251	0.3262	0.7920	0.7371	0.0031*	0.2371	0.0675	0.4296	0.0675	0.3571	0.4296	0.2929	0.8951	0.0505	0.0097*
	Holm-adj. p-value	0.0255*	0.4502	0.4502	1	1	0.0403*	1	0.675	4	0.675	1	1	1	1	0.5555	0.1164
	Estimate	-0.7040	-0.4000	-0.3040	-0.1000	0.1500	1.1500	0.4500	0.7000	0.3000	-0.7000	0.3500	0.3000	-0.4000	0.05000	0.7500	1.0000
	Standard Error	0.2612	0.3273	0.3079	0.3780	0.4453	0.3780	0.3780	0.3780	0.3780	0.3780	0.3780	0.3780	0.3780	0.3780	0.3780	0.3780
	DF	85.01	85	85.01	85	85	85	85	85	85	85	85	85	85	85	85	85
	t-Value	-2.70	-1.22	-0.99	-0.26	0.34	3.04	1.19	1.85	0.79	-1.85	0.93	0.79	-1.06	0.13	1.98	2.65
Subj. sleepiness (BT over sampling sessions)	Pr > |t|	0.9937	0.2116	0.1980	0.4936	0.8907	0.7211	0.1556	0.1244	0.5522	0.2857	0.4758	0.0469*	0.6342	0.1926	0.8119	0.3423
	Holm-adj. p-value	0.9937	0.594	0.594	1	1	1	1	1	1	1	1	0.6097	1	1	1	1
	Estimate	-0.00600	-0.4500	0.4440	-0.3000	-0.2500	0.1500	0.6000	0.6500	-0.2500	0.4500	0.3000	0.8500	0.2000	-0.5500	0.1000	0.4000
	Standard Error	0.7634	0.3575	0.3422	0.4363	1.8139	0.4188	0.4188	0.4188	0.4188	0.4188	0.4188	0.4216	0.4188	0.4188	0.4188	0.4188
	DF	84.6	84.98	85	85	81.7	85	85	85	85	85	85	85	85	85	85	85
	t-Value	-0.01	-1.26	1.30	-0.69	-0.14	0.36	1.43	1.55	-0.60	1.07	0.72	2.02	0.48	-1.31	0.24	0.96
Nocturnal sleep duration	Pr > |t|	0.1855	0.4537	0.8552	0.3028	0.1599	0.1173	0.2526	0.5932	0.3823	0.6911	0.8569	0.9573	0.6333	0.8166	0.7587	0.7296
	Holm-adj. p-value	0.5565	0.9074	0.9074	1	1	1	1	1	1	1	1	1	1	1	1	1
	Estimate	-17.4250	-14.3125	-3.1125	-20.8750	25.8500	33.0750	24.7500	-11.4250	18.6500	-8.3250	3.8750	-1.1000	10.3250	4.9750	-6.4500	7.2250
	Standard Error	13.0107	18.9982	16.9870	19.9051	17.4604	20.7864	21.4744	21.2845	21.1984	20.8397	21.4201	20.3869	21.5623	21.3783	20.8970	20.7955
	DF	60.28	71.65	69.43	29.6	14.6	55	78.8	67.2	64	55.7	73.6	37	84.8	72.4	56.6	55.1
	t-Value	-1.34	-0.75	-0.18	-1.05	1.48	1.59	1.15	-0.54	0.88	-0.40	0.18	-0.05	0.48	0.23	-0.31	0.35
Sleep efficiency	Pr > |t|	0.1855	0.4536	0.8554	0.3028	0.1602	0.1172	0.2525	0.5937	0.3822	0.6909	0.8569	0.9579	0.6333	0.8172	0.7587	0.7289
	Holm-adj. p-value	0.5565	0.9072	0.9072	1	1	1	1	1	1	1	1	1	1	1	1	1
	Estimate	-3.6301	-2.9825	-0.6476	-4.3500	5.3835	6.8925	5.1575	-2.3770	3.8860	-1.7350	0.8075	-0.2260	2.1510	1.0335	-1.3435	1.5090
	Standard Error	2.7104	3.9578	3.5394	4.1480	3.6389	4.3302	4.4743	4.4340	4.4160	4.3413	4.4623	4.2483	4.4924	4.4543	4.3532	4.3321
	DF	60.32	71.62	69.42	29.6	14.6	55.1	78.9	67.2	64	55.7	73.5	37	84.8	72.4	56.6	55.1
	t-Value	-1.34	-0.75	-0.18	-1.05	1.48	1.59	1.15	-0.54	0.88	-0.40	0.18	-0.05	0.48	0.23	-0.31	0.35
% of total sleep in N3	Pr > |t|	0.1305	<.0001*	0.0009*	0.0141*	0.9784	0.2380	0.0161*	0.6017	0.0961	0.0004*	<.0001*	0.0178*	0.0497*	0.0126*	0.0022*	0.2489
	Holm-adj. p-value	0.1305	0.0003*	0.0018*	0.1269	1	0.952	0.1288	1	0.4805	0.0048*	0.0013*	0.1288	0.2982	0.126	0.0242*	0.952
	Estimate Standard	2.2502	9.0430	-6.7928	-6.2430	0.0640	0.06400	-5.9215	-1.2285	3.9445	-8.7015	-12.1645	-5.9855	-4.7570	-6.1790	-7.4075	2.7160
	Error	1.4735	2.0375	1.9750	2.4621	2.3353	2.3353	2.4103	2.3434	2.3409	2.3372	2.3503	2.4653	2.3876	2.4234	2.3373	2.3372
	DF	83.16	65.98	83.85	55.3	19.8	19.8	83.5	75.3	75.1	74.9	76	68.2	80.6	84.7	74.9	74.9
	t-Value	1.53	4.44	-3.44	-2.54	0.03	0.03	-2.46	-0.52	1.69	-3.72	-5.18	-2.43	-1.99	-2.55	-3.17	1.16
% of total sleep in REM	Pr > |t|	0.2742	0.0154*	0.0808	0.6213	0.5808	0.1164	0.0203*	0.8007	0.1994	0.4014	0.0604	0.0741	0.0389*	0.9762	0.8240	0.3003
	Holm-adj. p-value	0.2742	0.0462*	0.1616	1	1	1	0.2639	1	1	1	0.6644	0.741	0.4668	1	1	1
	Estimate Standard	-1.1566	-3.6298	2.4731	-0.8585	0.9100	2.6505	4.0590	-0.4240	2.1645	1.4085	3.2005	3.1490	3.5730	0.05150	-0.3725	1.7405
	Error	1.0507	1.4610	1.3991	1.7280	1.6228	1.6689	1.7155	1.6739	1.6720	1.6690	1.6789	1.7358	1.7026	1.7224	1.6691	1.6689
	DF	82.66	68.03	83.3	54.9	21.4	75.5	84.2	76.1	75.8	75.5	76.9	67	81.7	85	75.5	75.5
	t-Value	-1.10	-2.48	1.77	-0.50	0.56	1.59	2.37	-0.25	1.29	0.84	1.91	1.81	2.10	0.03	-0.22	1.04

REM latency	Pr > |t|	0.2228	0.7634	0.5153	0.8477	0.4278	0.5328	0.8755	0.6504	0.8340	0.640	0.7206	0.4910	0.7971	0.2895	0.5388	0.8074
	Holm-adj. p-value	0.6684	1	1	1	1	1	1	1	1	1	1	1	1	1	1	1
	Estimate	-8.7392	-2.8250	-5.9142	2.2000	9.5250	6.8500	1.7250	-4.9750	2.3000	-5.1250	3.9250	-7.8000	-2.8250	11.7250	6.7500	-2.6750
	Standard Error	7.1140	9.3533	9.0526	11.4189	11.8970	10.9384	10.9772	10.9385	10.9384	10.9384	10.9391	11.2758	10.9531	11.0005	10.9384	10.9384
	DF	81.45	83.5	84.98	82	42.1	84.7	84.8	84.7	84.7	84.7	84.7	84.3	84.7	84.9	84.7	84.7
	t-Value	-1.23	-0.30	-0.65	0.19	0.80	0.63	0.16	-0.45	0.21	-0.476	0.36	-0.69	-0.26	1.07	0.62	-0.24
Daytime sleep duration	Pr > |t|	0.0023*	0.0768	0.5220	0.2078	0.0360*	0.7921	0.3619	0.7253	0.1000	0.5160	0.0316*	0.1764	0.3151	0.0006*	0.002*	0.047*
	Holm-adj. p-value	0.0069*	0.1536	0.522	1	0.360	1	1	1	0.8	1	0.3476	1	1	0.0078*	0.024*	0.423
	Estimate	8.8277	6.6000	2.2277	-5.4000	-9.7000	-1.1250	-3.9000	1.5000	7.0750	-2.7750	-9.3000	5.8000	4.3000	-15.1000	-13.6000	8.5750
	Standard Error	2.8135	3.6846	3.4654	4.2548	4.5524	4.2547	4.2547	4.2547	4.2547	4.2547	4.2547	4.2547	4.2547	4.2547	4.2547	4.2547
	DF	85.02	85	85.01	85	85	85	85	85	85	85	85	85	85	85	85	85
	t-Value	3.14	1.79	0.64	-1.27	-2.13	-0.26	-0.92	0.35	1.66	-0.65	-2.19	1.36	1.01	-3.55	-3.20	2.02

BDC: baseline data collection; HDT: head-down tilt bed rest; R: recovery. BDC1: first sampling day during BDC, performed during day -12/-11; BDC2: second sampling day during BDC, performed during day -4/-3; HDT1: first sampling day during HDT, performed during day 1/2; HDT2: second sampling day during HDT, performed during day 26/27; HDT3: third sampling day during HDT, performed during day 53/54; R: sampling day during R, performed during day +10+11. Pr > |t|: nominal p-value; Holm-adj. p-value: p-value adjusted by Holm-Bonferroni correction. DF: degrees of freedom.

Supplementary Figures

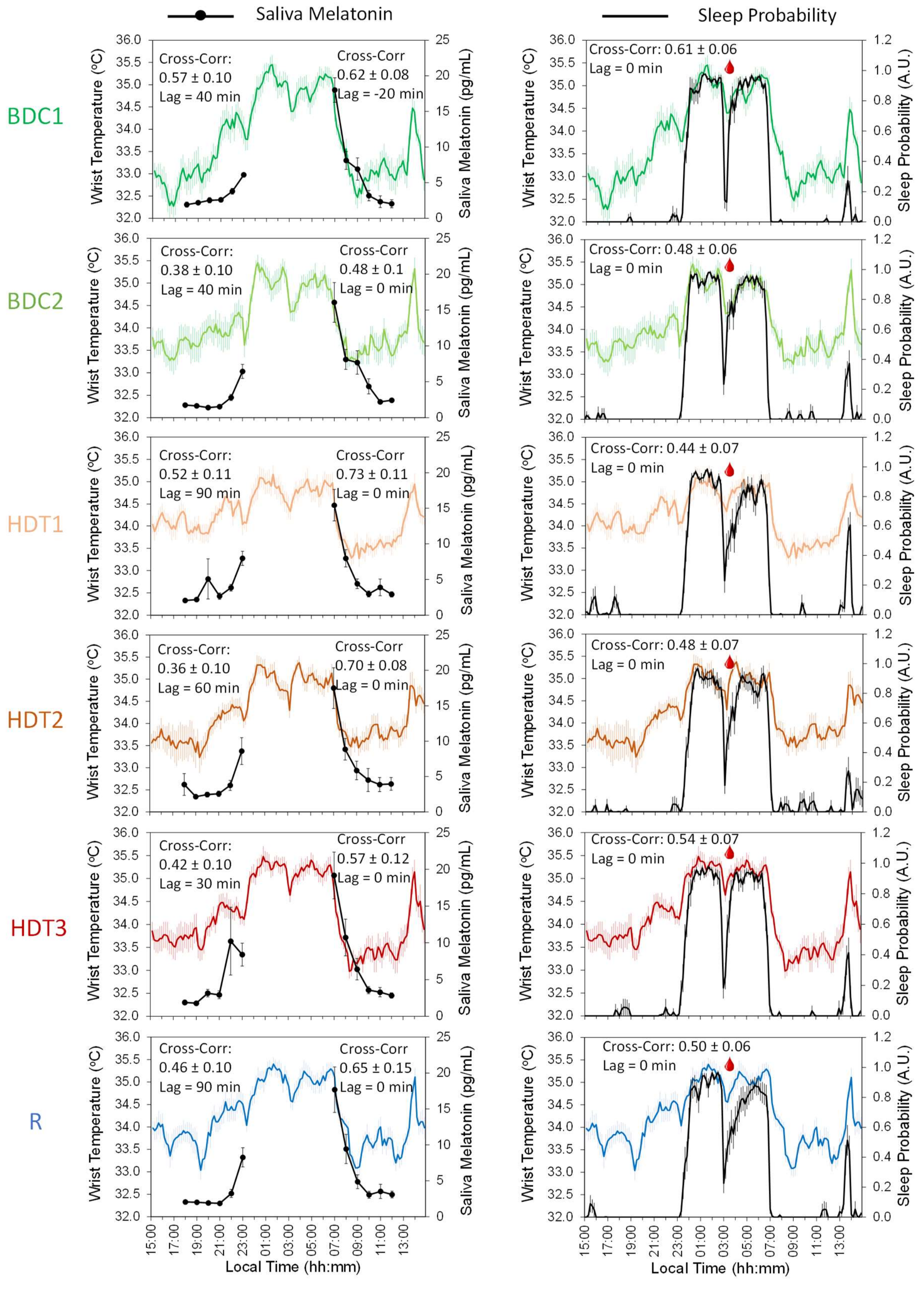

Supplementary Figure 1. Wrist skin temperature and its correlation with saliva melatonin (left) and sleep probability (right). Average (± SEM) wrist skin temperature during the sampling sessions (BDC1, dark green; BDC2, light green; HDT1, pink; HDT2, orange; HDT3, red; R, blue) and comparison with average (± SEM) saliva melatonin profile (left panel, black line and circles), and sleep probability (right panel, black line). Average (± SEM) cross-correlation coefficients and lags indicate the degree of coincidence between wrist temperature vs. melatonin (rising part, left; declining part, right) and sleep probability across the day. Red drop indicates blood sampling at night.

a

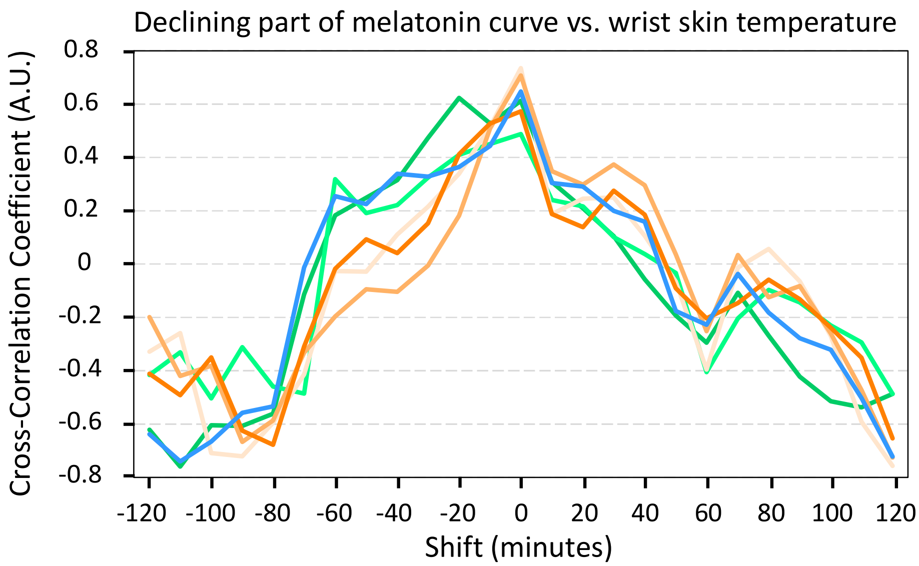
Declining part of melatonin curve vs. wrist skin temperature
Cross-Correlation Coefficient (A.U.)
0.8
0.6
0.4
0.2
0
-0.2
-0.4
-0.6
-0.8
-120 -100 -80 -60 -40 -20 0 20 40 60 80 100 120
Shift (minutes)

b

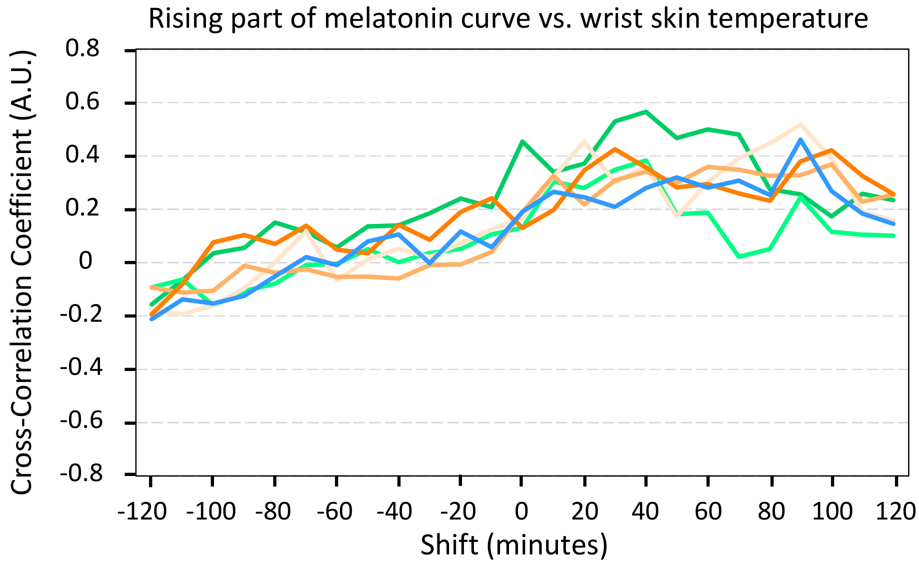
Rising part of melatonin curve vs. wrist skin temperature
Cross-Correlation Coefficient (A.U.)
0.8
0.6
0.4
0.2
0
-0.2
-0.4
-0.6
-0.8
-120 -100 -80 -60 -40 -20 0 20 40 60 80 100 120
Shift (minutes)

c

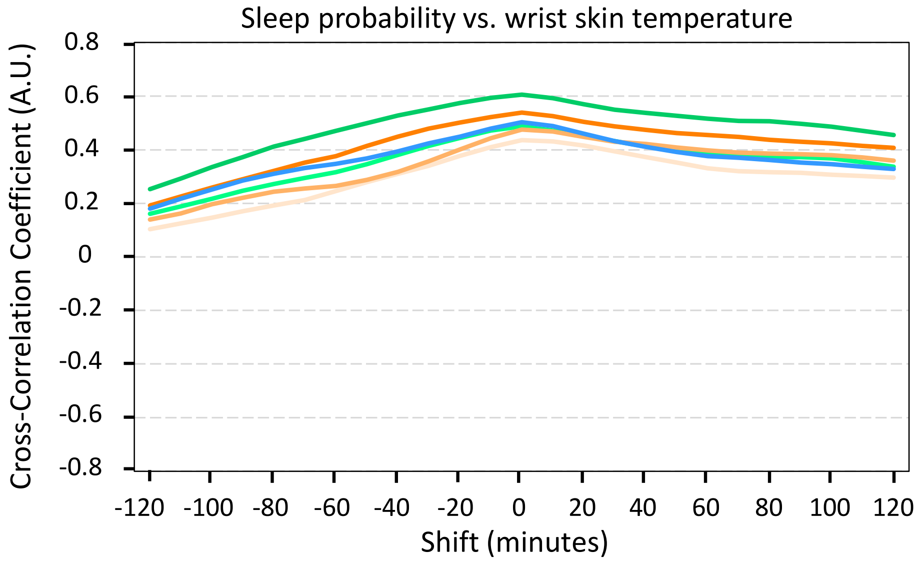
Sleep probability vs. wrist skin temperature
Cross-Correlation Coefficient (A.U.)
0.8
0.6
0.4
0.2
0
-0.2
-0.4
-0.6
-0.8
-120 -100 -80 -60 -40 -20 0 20 40 60 80 100 120
Shift (minutes)

Supplementary Figure 2. Cross-correlations between rising (A) and declining (B) part of the melatonin profile, and sleep probability (C) vs. wrist skin temperature (BDC1, dark green; BDC2, light green; HDT1, pink; HDT2, orange; HDT3, red; R, blue). Error bars have been omitted for clarity (N=20). Positive lags indicate wrist skin temperature profile advanced with respect to the other variables.

Supplementary Notes

The study was divided in two campaigns (each enrolling 10 participants), the first occurring January-April 2017, and the second in September-December 2017 (see Methods section for further details). The campaign effects could, thus, reflect a possible seasonal effect.

Regarding participant light exposure measured by wrist-worn actiwatches, when considering both campaigns separately, there was a significant interaction between campaign and segment ($p = 0.0011$). Although no significant differences were found between campaigns during R, higher levels of light exposure were measured during campaign 2 in BDC (83.97 ± 8.58 lux) and HDT (67.40 ± 7.67 lux) vs. campaign 1 (BDC: 62.38 ± 4.82 lux, $p = 0.033$; HDT: 42.34 ± 5.23 lux, $p = 0.0144$) (Supplementary Figure 3a). In campaign 1, higher levels of light were present both in BDC (62.38 ± 4.82 lux, $p_{adj} = 0.0176$) and R (78.02 ± 7.62 lux, $p_{adj} = 0.0003$) when compared to HDT (42.34 ± 5.23 lux). During R, light levels were significantly higher than during BDC ($p_{adj} = 0.0371$). In C2, levels were found to be similar during HDT (67.40 ± 7.67 lux) compared to BDC (83.98 ± 8.58 lux, $p_{adj} = 0.0556$) and R (65.41 ± 6.93 lux, $p_{adj} = 0.7836$). During BDC, higher levels of light exposure were recorded when compared to R ($p_{adj} = 0.0435$).

Supplementary Figure 4 shows the differences in natural photoperiod during the course of campaign 1 and 2, produced by different sunrise and sunset times (Supplementary Figure 4a), which lead to different daylengths (Supplementary Figure 4b). At the beginning, campaign 1 occurred under shorter natural photoperiods (more than 2 hours shorter), while at the end, campaign 1 daylight was up to 4 hours longer than at the end of campaign 2.

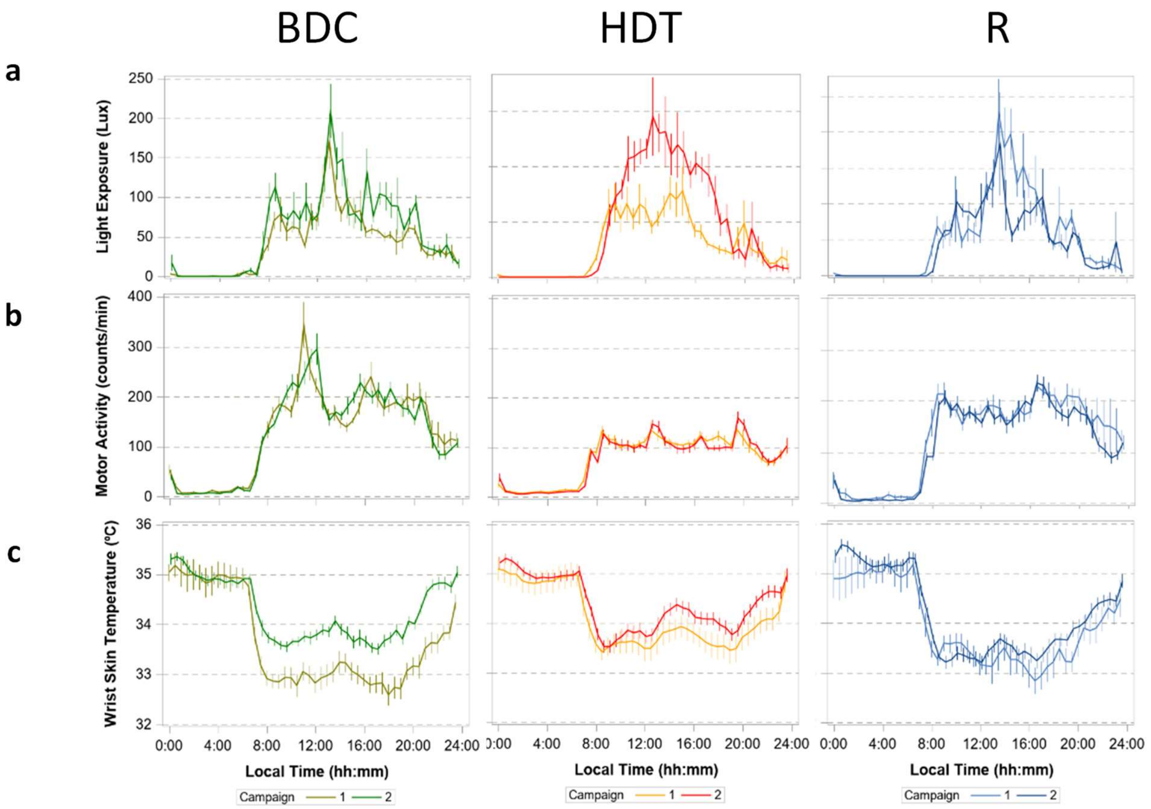

Supplementary Figure 3. Effect of the campaign. Effect of campaign on average white light exposure (a), average motor activity (b), and average wrist skin temperature (c) during each protocol segment. Campaign 1 January – April, campaign 2 September – December.

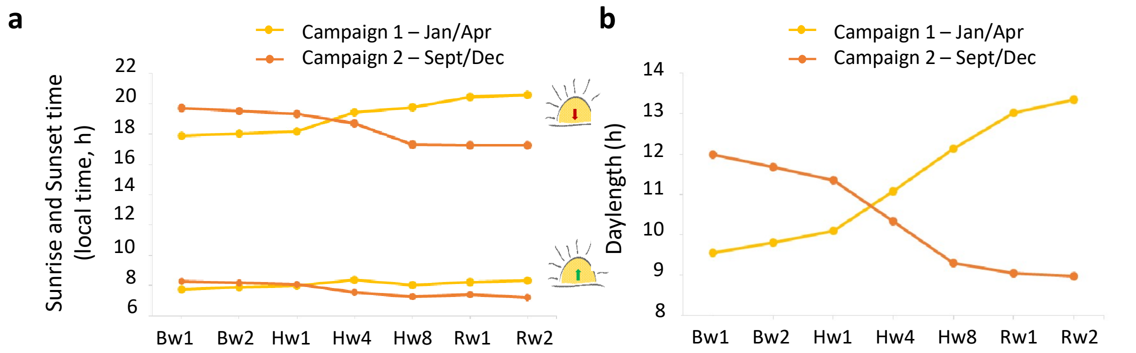

Supplementary Figure 4. Sunrise/sunset times (a) and daylength (b) over campaigns 1 (January – April, yellow) and 2 (September–December, orange), by weeks over segments (Bw1: first week of BDC; Bw2: second week of baseline data collection, BDC; Hw1: first week of head down tilt bed rest, HDT; Hw4: fourth week of head down tilt bed rest; Hw8: eighth week of head down tilt bed rest; Rw1: First week of recovery segment, R; Rw2: Second week of recovery segment).

Activity levels during the day did not show any significant differences between campaigns, whereas activity during the night showed an interaction between campaign and segment ($p = 0.0146$), with lower night-time levels during campaign 2 in R (7.35 ± 0.60) than in BDC (8.76 ± 0.59, $p_{adj} = 0.0268$) or HDT (9.21 ± 0.73, $p_{adj} = 0.0084$), while no differences between segments were found in campaign 1 (Supplementary Figure 3b).

When considering both campaigns separately for daytime, there was a significant interaction between campaign and segment ($p < 0.0001$). In campaign 1, daytime WST was higher in recovery (33.29 ± 0.22 ºC) than baseline (32.92 ± 0.18) ($p_{adj} = 0.0006$), while the opposite occurred in campaign 2 (BDC 33.74 ± 0.09 ºC, R 33.45 ± 0.11 ºC; $p_{adj} = 0.005$). This interaction was also reflected in amplitude ($p = 0.0001$), with lower and greater amplitudes in recovery than baseline in campaign 1 (BDC: 2.03 ± 0.35 ºC

vs R: 1.73 ± 0.32 ºC, p_{adj} = 0.0290) and 2 (BDC: 1.23 ± 0.08 ºC vs R: 1.82 ± 0.16 ºC, p_{adj} = 0.0003), respectively (Supplementary Figure 3c). A clear seasonal effect was detected in wrist skin temperature amplitude during BDC, with greater amplitudes during C1, in (2.03 ± 0.35 ºC) than during C2 (1.23 ± 0.08 ºC) (p = 0.0399), which was abolished during HDT and R (p > 0.05).

The scheduling of campaigns had a significant effect on the onset of melatonin rhythms (Table S5) reflected by an interaction between campaign and sampling session (p = 0.0053). In campaign 1 (C1, Jan-Apr), there was a slight tendency to a phase advance from the second baseline session (BDC2, 22.42 ± 0.63 h) towards the second (HDT2, 21.53 ± 0.39 h) (p_{adj} = 0.5434) and third sessions of bed rest (HDT3, 21.32 ± 0.26 h) (p_{adj} = 0.1965). During recovery (R, 22.40 ± 0.44 h), there was a trend to delay in melatonin compared to the last two sessions of bed rest (HDT2, p_{adj} = 0.5772; HDT3, p_{adj} = 0.2142). In campaign 2, however, melatonin onset showed a progressive delay from the beginning of bed rest (HDT1, 20.93 ± 0.23) towards the end (HDT3, 22.38 ± 0.32, p_{adj} = 0.0195). Although it tended to show an advance at the beginning of bed rest with respect to baseline (p_{adj} > 0.05), melatonin onset was tended to delay at the end of bed rest (HDT3, 22.38 ± 0.32) with respect to the beginning of baseline (BDC1, 21.36 ± 0.29) (p_{adj} = 0.270). Recovery (R), however, tended to delay with respect to the beginning of bed rest (HDT1) (p_{adj} = 0.2314), although tending to advance with respect to the end of bed rest (HDT2, p_{adj} = 1; and HDT3, p_{adj} = 1).

Supplementary Table 5. Melatonin onset and offset through sampling sessions and campaigns.

Sampling session	MOn (h) (campaigns combined)	MOn C1 (h)	MOn C2 (h)	MOff (h) (campaigns combined)	MOff C1 (h)	MOff C2 (h)
BDC1	21.70 ± 0.27	22.05 ± 0.44	21.36 ± 0.29	09.74 ± 0.28	09.16 ± 0.40	10.33 ± 0.30
BDC2	22.02 ± 0.34	22.42 ± 0.63	21.61 ± 0.23	11.81 ± 1.31	12.58 ± 3.07	11.20 ± 0.29
HDT1	21.28 ± 0.15	21.62 ± 0.12	20.93 ± 0.23	10.01 ± 0.36	09.27 ± 0.32	10.83 ± 0.56
HDT2	21.85 ± 0.24	21.53 ± 0.39	22.17 ± 0.25	09.62 ± 0.36	08.86 ± 0.32	10.38 ± 0.56
HDT3	21.85 ± 0.23	21.32 ± 0.26	22.38 ± 0.32	10.56 ± 0.76	11.05 ± 1.62	10.13 ± 0.29
R1	22.20 ± 0.24	22.40 ± 0.44	22.01 ± 0.20	10.93 ± 1.11	10.42 ± 2.38	11.39 ± 0.35

Melatonin onset (MOn) and offset (MOff) across the different sampling sessions, combined and split by campaign (C1, from January to April; C2, from September to December).

There was a clear effect of campaign on average saliva melatonin concentrations (07:00 – 12:00 and 18:00 – 23:00), with higher saliva concentrations during the second campaign (5.62 ± 0.30 pg/mL) than during the first (3.77 ± 0.32 pg/mL) ($p = 0.0007$) (Supplementary Figure 5). When considering morning (07:00 – 12:00) and evening (18:00 – 23:00) melatonin concentrations, this difference between campaigns is maintained ($p = 0.0009$ and $p = 0.0028$, respectively) (for specific melatonin concentrations by campaign and sampling session, see Table S6). Only evening concentration at HDT2 was statistically similar ($p = 0.097$) between both campaigns. With that exception, averaged, evening and morning saliva melatonin concentrations were higher in C2 than in C1 in all sampling sessions ($p < 0.022$).

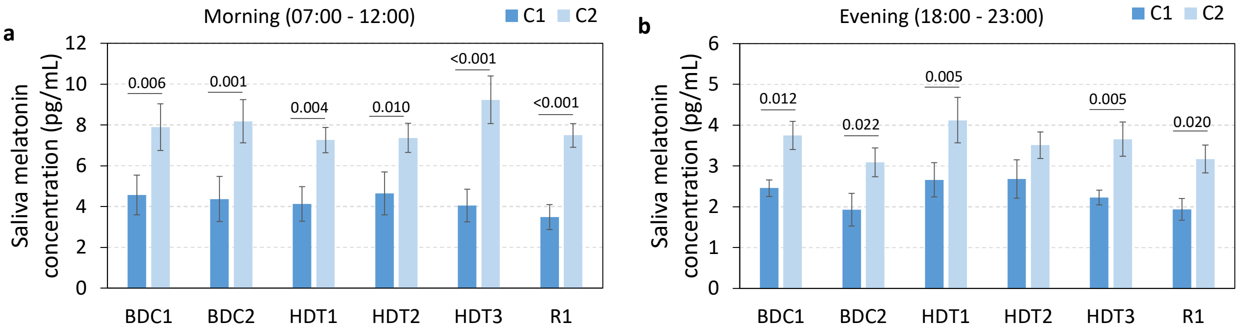

Supplementary Figure 5. Effect of campaign on saliva melatonin concentration. Comparison of average (± SEM) melatonin concentrations between campaigns in each protocol session for morning (a, 07:00 – 12:00) and evening (b, 18:00 – 23:00) samples. P values for comparisons are listed.

Supplementary Table 6. Melatonin concentrations (averaged, evening and morning) by sampling and campaign.

	Averaged melatonin concentration (pg/mL)		Evening melatonin concentration (pg/mL)		Morning melatonin concentration (pg/mL)	
	C1	C2	C1	C2	C1	C2
BDC1	3.35 ± 0.51	5.32 ± 0.58	2.46 ± 0.20	3.75 ± 0.35	4.57 ± 0.97	7.89 ± 1.15
BDC2	3.39 ± 0.65	5.21 ± 0.59	1.93 ± 0.40	3.09 ± 0.36	4.37 ± 1.11	8.18 ± 1.06
HDT1	3.67 ± 0.72	5.47 ± 0.64	2.66 ± 0.42	4.12 ± 0.56	4.13 ± 0.85	7.26 ± 0.62
HDT2	4.31 ± 1.01	5.86 ± 0.84	2.67 ± 0.47	3.51 ± 0.33	4.65 ± 1.05	7.37 ± 0.71
HDT3	4.34 ± 0.98	6.45 ± 1.00	2.23 ± 0.18	3.66 ± 0.42	4.05 ± 0.8	9.23 ± 1.17
R1	3.53 ± 0.87	5.40 ± 0.78	1.94 ± 0.26	3.17 ± 0.34	3.48 ± 0.60	7.49 ± 0.58

Averaged, evening (18:00 – 23:00) and morning saliva melatonin (07:00 – 12:00) concentration for each sampling session and campaign (C1, from January to April; C2, from September to December).

Subjective sleepiness at breakfast was significantly higher during all segments of campaign 2 than campaign 1 ($p = 0.0057$). This could be due to the previously described seasonal effect on sleepiness in the morning.

Overall, average nocturnal sleep duration was longer during campaign 2 (6.07 ± 0.08 h) compared with 1 (5.75 ± 0.11 h) ($p = 0.0326$). Regarding differences between campaigns per sampling session, in BDC1 ($p = 0.0387$) and HDT3 ($p = 0.0114$), sleep duration was significantly longer during campaign 2 (BDC1, 6.29 ± 0.11 h; HDT3, 6.21 ± 0.13 h) than during campaign 1 (BDC1, 5.79 ± 0.22 h; HDT3, 6.09 ± 0.13 h), respectively.

An important aim of this project was to confirm the effectiveness of an anti-inflammatory cocktail as a countermeasure for microgravity, to be used by astronauts to alleviate adverse effects of microgravity. Regarding the effects of this cocktail in our biological rhythms and sleep, it seemed to have an effect only on mean levels of melatonin and cortisol.

Melatonin levels were lower in the group treated with cocktail (3.75 ± 0.23 pg/mL) than in controls (5.24 ± 0.28 pg/mL) ($p = 0.0223$), but no interaction between group and sampling session was found. Since these differences were already present in BDC before the start of the treatment they probably do not represent an effect of treatment.

Cortisol levels were, in general, lower, in the group treated with cocktail (6.36 ± 0.21 nmol/L) than in control (8.29 ± 0.22 nmol/L) ($p = 0.0025$), but no interaction between group and sampling session was found.

There was no significant difference in subjective sleepiness between control and cocktail groups when considering the whole study. However, at HDT1 the KSS score

was significantly lower in the cocktail (3.8 ± 0.30) than in the control group (5.2 ± 0.29) (p = 0.0032) although this was measured on the first day of HDT and cocktail treatment administration so this difference may not be meaningful.

Supplementary Table 7. Sleep habits before entering the study, according to the Munich Chronotype Questionnaire:

	**Bed Time (local time, hh:mm) mean ± SD**	**Wake-up time (local time, hh:mm) mean ± SD**
**Work Days**	23:08 ± 00:44	07:10 ± 01:02
**Free Days**	00:00 ± 01:05	8:26 ± 01:06

Supplementary Discussion

Apart from the effects of the bed rest protocol itself, we also detected some seasonal effects, evidenced by different campaign scheduling. The amplitude or wrist skin temperature was greater during the first two weeks of campaign 1 (end of January/beginning of February) than during the first two weeks of campaign 2 (end of September/beginning of October). These results are in line with previous published data on distal skin temperature that also exhibited greater amplitudes in winter than in summer [1]. Saliva melatonin concentrations were apparently affected by season, with higher levels in the second (Sept-Dec) compared with the first campaign (Jan-Apr). Seasonal differences in melatonin concentration have been previously described, being higher in summer than in winter [2]. However, and surprisingly, these differences

were not attenuated throughout the progress of the study. By contrast, at the end of both campaigns (during recovery), occurring in April (C1) and December (C2), these differences remained practically the same as at the beginning of the study. Although we cannot discard a confounding effect due to a possible shift in melatonin phase (since our protocol did not allow to capture the maximum melatonin concentrations) or different light exposures, this 'memory' effect could also be related to the fact that the participants remained in a constant environment when they entered the clinic, which may have stabilised the melatonin concentration levels throughout the study, thus abolishing the expected inversion [i.e., lower levels at the end of C2, occurring in December (Winter) than at the end of C1, occurring in April (Spring)].

We also observed a seasonal effect on parameters related to sleep, with longer nocturnal sleep durations during the second (Sept-Dec) than during the first campaign (Jan-Apr). Subjective sleepiness before breakfast was also higher in the first campaign, reflecting a greater need of sleep in Autumn than Winter/Spring. This is in accordance with previous studies (not in bed rest conditions) where sleep duration has been found to increase as daylength gets longer [3–9]. O'Connell et al., however, failed to find this seasonal effect in sleep [10].

Supplementary References

1. Martinez-Nicolas, A. *et al.* Daytime variation in ambient temperature affects skin temperatures and blood pressure: Ambulatory winter/summer comparison in healthy young women. *Physiol. Behav.* **149**, 203–211 (2015).
2. Münch, M., Ladaique, M., Roemer, S., Hashemi, K. & Kawasaki, A. Melanopsin-mediated acute light responses measured in winter and in summer: Seasonal variations in adults with and without cataracts. *Front. Neurol.* **8**, 464 (2017).
3. De La Iglesia, H. O. *et al.* Access to electric light is associated with shorter sleep duration in a traditionally hunter-gatherer community. *J. Biol. Rhythms* **30**, 342–350 (2015).
4. Lehnkering, H. & Siegmund, R. Influence of chronotype, season, and sex of subject on sleep behavior of young adults. *Chronobiol. Int.* **24**, 875–88 (2007).
5. Cepeda, M. *et al.* Seasonality of physical activity, sedentary behavior, and sleep in a middle-aged and elderly population: The Rotterdam study. *Maturitas* **110**, 41–50 (2018).
6. Hjorth, M. F. *et al.* Seasonal variation in objectively measured physical activity, sedentary time, cardio-respiratory fitness and sleep duration among 8-11 year-old Danish children: a repeated-measures study. *BMC Public Health* **13**, 808 (2013).
7. Thorleifsdottir, B., Björnsson, J. K., Benediktsdottir, B., Gislason, T. & Kristbjarnarson, H. Sleep and sleep habits from childhood to young adulthood over a 10-year period. *J. Psychosom. Res.* **53**, 529–537 (2002).
8. Carskadon, M. A. & Acebo, C. Parental reports of seasonal mood and behavior changes in children. *J. Am. Acad. Child Adolesc. Psychiatry* **32**, 264–269 (1993).

9. Robbins, R. *et al.* Four-year trends in sleep duration and quality: A longitudinal study using data from a commercially available sleep tracker. *J. Med. Internet Res.* **22**, e14735 (2020).

10. O'Connell, S. E., Griffiths, P. L. & Clemes, S. A. Seasonal variation in physical activity, sedentary behaviour and sleep in a sample of UK adults. *Ann. Hum. Biol.* **41**, 1–8 (2014).
